# Efficiency of ITS Sequences for DNA Barcoding in *Passiflora* (Passifloraceae)

**DOI:** 10.3390/ijms16047289

**Published:** 2015-04-01

**Authors:** Giovanna Câmara Giudicelli, Geraldo Mäder, Loreta Brandão de Freitas

**Affiliations:** Laboratory of Molecular Evolution, Department of Genetics, Universidade Federal do Rio Grande do Sul, P.O. Box 15053, 91501-970 Porto Alegre, Brazil; E-Mails: gigiudicelli@hotmail.com (G.C.G.); geraldo.mader@gmail.com (G.M.)

**Keywords:** rDNA internal transcribed spacer, plant DNA barcoding, phylogenetic signal, *Passiflora*

## Abstract

DNA barcoding is a technique for discriminating and identifying species using short, variable, and standardized DNA regions. Here, we tested for the first time the performance of plastid and nuclear regions as DNA barcodes in *Passiflora*. This genus is a largely variable, with more than 900 species of high ecological, commercial, and ornamental importance. We analyzed 1034 accessions of 222 species representing the four subgenera of *Passiflora* and evaluated the effectiveness of five plastid regions and three nuclear datasets currently employed as DNA barcodes in plants using barcoding gap, applied similarity-, and tree-based methods. The plastid regions were able to identify less than 45% of species, whereas the nuclear datasets were efficient for more than 50% using “best match” and “best close match” methods of TaxonDNA software. All subgenera presented higher interspecific pairwise distances and did not fully overlap with the intraspecific distance, and similarity-based methods showed better results than tree-based methods. The nuclear ribosomal internal transcribed spacer 1 (ITS1) region presented a higher discrimination power than the other datasets and also showed other desirable characteristics as a DNA barcode for this genus. Therefore, we suggest that this region should be used as a starting point to identify *Passiflora* species.

## 1. Introduction

DNA barcoding is a method that involves species identification and discrimination using short, variable, and standardized DNA regions [1,2]. A DNA sequence is considered to be helpful as a barcode when it conforms to three basic criteria: (i) meaningful genetic variability at the species level to enable species discrimination; (ii) a short sequence length to facilitate DNA extraction and amplification; and (iii) conserved flanking regions for the development of universal primers across highly divergent taxa [3,4,5].

In animal genomes, the most accepted sequence used as a DNA barcode is the mitochondrial cytochrome oxidase I gene (COI). However, studies in plants show that the insufficient variability of this region caused by its low mutation rate, has led to the search for alternative barcoding regions [3,6,7]. As a result, many different plastid loci and combinations of these loci have been proposed as promising DNA barcoding in plants [3,8]. In studies comparing different markers, some observed that each group presents distinct plastid loci or combinations of loci as an ideal barcode [9,10,11,12], whereas others highlight the challenges with the use of plastid data for some groups [13,14,15]. Therefore, many researchers have accepted that multiple markers may be necessary to obtain appropriate species discrimination [16,17].

In addition to plastid markers, the nuclear ribosomal internal transcribed spacer (ITS) region has also been indicated as a barcoding region [3,6,18,19,20]. Despite the problems associated with this marker [21,22], it has been shown to perform better when compared with either coding or noncoding plastid markers [23,24,25,26,27,28]. Many studies have also compared the discriminatory power revealed by the ITS region in its entirety with ITS2 [29,30,31,32], proposing the use of ITS2 as an alternative barcode to the entire ITS region due to the difficulty in amplifying and directly sequencing the entire region. In spite of this, the ITS1 region has rarely been tested as a DNA barcode in plants [33]. Comparisons between ITS1 and ITS2 in 10 major groups of eukaryotes suggest that ITS1 represents a better barcode than ITS2 for eukaryotic species [34].

*Passiflora* L., the largest genus in Passifloraceae, comprises more than 520 species largely distributed in the Neotropical region [35,36], with just a few species occurring in the Old World [37]. The wide diversity of floral and vegetative features contributes to the large diversity and complex taxonomy of this genus [38].

The *Passiflora* genus was initially divided into 22 [39] or 23 [40] subgenera based on floral morphology. The current infrageneric taxonomy [41] regrouped the species into four subgenera: *Astrophea* (DC.) Mast, *Decaloba* (DC.) Rchb, *Deidamioides* (Harms) Killip, and *Passiflora*. Subsequent phylogenetic studies performed using distinct molecular markers and different amounts and proportions of species recovered well-supported clades corresponding partially [42] or fully [43,44,45,46] to this infrageneric classification.

Despite the ecological and economic importance of *Passiflora* species, molecular markers have only recently been utilized in genetic studies of this genus. In addition, both basic genetic researches related to population studies and pre-breeding programs remain scarce for most *Passiflora* species (for a review, see [47]). Considering the number of *Passiflora* species and the increasing use of these species as a resource for ornamental, medicinal, and food purposes, a simple source of genetic markers to identify the different species is necessary.

Several studies in *Passiflora* have been conducted utilizing the ITS region for different proposes [36,38,43,48,49,50,51]. These studies demonstrate the phylogenetic signal of ITS in *Passiflora* and the subsequent contribution of this marker in clarifying the evolutionary relationships between and within species of the genus. Although the results were not based on the DNA barcoding concept, they did indicate a potential role for the ITS region in resolving species identification and differentiation in *Passiflora*.

In this study, we evaluate the potential utility of ITS regions for identifying and discriminating *Passiflora* species based on a representative sample consisting of approximately 40% of the genus. The applicability and effectiveness of different regions (ITS1 and ITS2) in discriminating species across *Passiflora* were studied for the first time. Because the plastid genes *rbcL* and *matK* have been suggested as the standard barcode for land plants [5,8], sequences of these markers available in GenBank were also tested as candidates for DNA barcodes in *Passiflora*, as were other markers commonly used in barcoding studies with sufficient sequences available in GenBank for this analysis, such as *trnH-psbA* and the *trnL* (UAA) intron [52]. The main goals of this study were as follows: (i) to test different standard barcode regions in *Passiflora*; (ii) to compare the effectiveness of the ITS1, ITS2, and ITS1+2 regions as barcoding candidates for *Passiflora*, selecting the region most suitable for distinguishing species in this genus; and (iii) to compare different methods of evaluating barcodes in plants.

## 2. Results

### 2.1. Sequence Characteristics

The results for analyses of *rbcL*, *matK*, *trnH-psbA*, and the *trnL* (UAA) intron showed that these markers present low interspecific variability in *Passiflora* (Supplementary Table S1). Indeed, they were only able to identify less than 45% of *Passiflora* species using the TAXONDNA software and criteria previously described, and they also presented low discrimination power between subgenera. Based on these results, these markers sequences were not included in our further analyses.

The sequence characteristics of the ITS regions evaluated in this study are summarized in Table 1. The ITS1 alignment length was always greater than that of ITS2 within each subgenus. The subgenus *Decaloba* presented the longest alignment length for both datasets, whereas shorter alignment lengths for ITS1 and ITS2 were observed in subgenera *Astrophea* and *Deidamioides*, respectively. *Decaloba* also had the highest percentage of variable and informative sites, in addition to the highest overall Kimura-2-Parameters distance (K2P) compared to the other subgenera. *Astrophea* showed lowers values of variable and informative characters, whereas *Deidamioides* (ITS1 and ITS1+2) and *Passiflora* (ITS2) presented lower overall K2P distances. ITS1 commonly presented a higher percentage of variable and informative sites compared to ITS2, except for *Deidamioides*.

**Table 1:** Characteristics of each internal transcribed spacer (ITS) dataset presented per subgenus.

Subgenus	Barcode Region	*N* Individuals	*N* Species	*N* Singletons	Alignment Length (bp)	Variable Characters(%)	PI Characters (%)	Overall K2P (%)
*Astrophea*	ITS1	53	16	12	291	32.99	22.68	8.8
	ITS2	53	16	12	237	28.27	16.46	7.2
	ITS1+2	53	16	12	528	30.87	19.89	8.0
*Decaloba*	ITS1	314	134	85	359	76.88	65.46	24.7
	ITS2	314	134	85	258	72.87	56.59	14.0
	ITS1+2	314	134	85	617	75.20	61.75	19.7
*Deidamioides*	ITS1	101	8	3	301	40.53	24.92	4.8
	ITS2	101	8	3	226	44.25	25.22	5.8
	ITS1+2	101	8	3	527	42.13	25.05	5.3
*Passiflora*	ITS1	287	64	46	292	55.48	39.73	8.8
	ITS2	287	64	46	249	52.21	30.92	3.8
	ITS1+2	287	64	46	541	53.97	35.67	6.5

ITS, ribosomal DNA internal transcribed spacer; BP, base pairs; PI, parsimony informative; K2P, pairwise genetic distance Kimura-2-Parameters.

### 2.2. Assessment of Barcoding Gap

**Figure 1:**
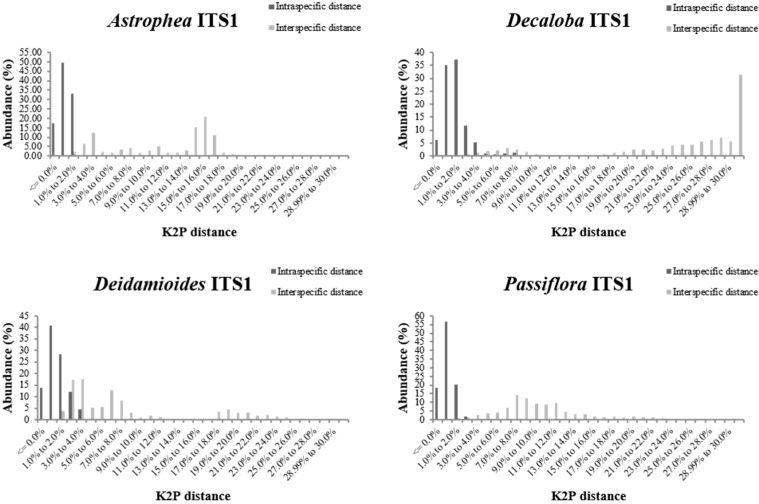
Relative abundance of intra- and inter-specific Kimura-2-Parameter pairwise distance considering the ITS1 dataset in subgenera *Astrophea*, *Decaloba*, *Deidamioides*, and *Passiflora*.

The relative distribution of the frequencies of K2P distances was calculated for the three ITS datasets for all *Passiflora* subgenera using TAXONDNA software, and the pairwise intra- and inter-specific genetic distances showed a similar pattern for all subgenera and datasets. To illustrate the observed patterns, the ITS1 results are shown in Figure 1, and the results for ITS2 and ITS1+2 are presented in Figure S1 and Figure S2. The interspecific distance was higher in all subgenera and did not fully overlap with the intraspecific distance. Therefore, the barcoding gap was identified for all datasets and subgenera.

### 2.3. “Best Match” and “Best Close Match” Analyses

The results of similarity tests performed in TAXONDNA software are shown in Table 2. In the subgenus *Astrophea*, the same success rate of species identification (74%) was observed for the three datasets based on both TAXONDNA functions: BM and BCM. The other subgenera presented higher values of correct identification when BM was selected compared to BCM. The lowest discriminatory powers were obtained using ITS2 in the subgenera *Decaloba* (BM: 51%; BCM: 50%) and *Passiflora* (BM: 55%; BCM: 51%); nevertheless, more than 50% of species were correctly identified. The three datasets recovered the same percentage of correctly identified species in subgenera *Astrophea* (BM and BCM: 74%) and *Deidamioides* (BM: 96%; BCM: 95%); in contrast, ITS1+2 showed the best results in the subgenus *Decaloba* (BM: 65%; BCM: 64%), and ITS1 performed better in the subgenus *Passiflora* (BM: 82%; BCM: 78%). The highest rates of correct identification were observed in *Deidamioides* and the lowest values in *Decaloba*. Comparing the results of the BM and BCM options, we observed that BCM presented a lower discriminatory power than BM, most likely because BCM is a more stringent analysis.

**Table 2:** DNA barcoding performance evaluated based on similarity methods per ITS dataset per subgenus.

Subgenus	Barcode Region	*N* Individuals	BM (%)			BCM (%)				Threshold (%)
			Correct	Ambiguous	Incorrect	Correct	Ambiguous	Incorrect	No Match	
*Astrophea*	ITS1	53	73.58	5.66	20.75	73.58	3.77	5.66	16.98	1.51
	ITS2	53	73.58	3.77	22.64	73.58	3.77	9.43	13.20	2.97
	ITS1+2	53	73.58	3.77	22.64	73.58	3.77	5.66	16.98	1.92
*Decaloba*	ITS1	314	63.05	4.13	32.80	61.78	3.82	23.24	11.14	3.77
	ITS2	314	50.95	16.87	32.16	50.31	16.55	25.15	7.96	3.46
	ITS1+2	314	64.64	1.27	34.07	64.01	1.27	23.88	10.82	3.43
*Deidamioides*	ITS1	101	96.03	0	4.96	95.04	0	0	4.95	2.98
	ITS2	101	96.03	0	4.96	95.04	0	0	4.95	5.41
	ITS1+2	101	96.03	0	4.96	95.04	0	0	4.95	3.56
*Passiflora*	ITS1	287	81.53	1.74	16.72	78.39	1.39	5.57	14.63	1.83
	ITS2	287	54.70	28.57	16.72	50.87	27.87	9.40	11.84	1.19
	ITS1+2	287	81.18	1.74	17.07	77.00	1.39	4.52	17.07	1.28

BM, best match, and BCM, best close match (according to [53]) obtained in TaxonDNA software.

### 2.4. Tree-Based Methods

The evaluation of barcode sequences based on phylogenetic trees was estimated according to the correct assignment of individuals (Table 3) in their respective subgenus or species group, respectively. Considering the phylogenetic method, BI recovered the highest value of species monophyly, except for ITS2 in *Deidamioides*, for which NJ performed better (63% of species correctly identified); this last result was due to the identification of one extra species with the NJ method compared to BI. Comparing the datasets within each subgenus and tree-based method, the highest discriminatory power was observed when ITS1+2 was used in all cases, except for *Deidamioides*. In this subgenus, ITS2 performed better using a NJ approach, and ITS1 recovered the same percentage of correctly identified species as ITS1+2 using the ML method. Although BI performed slightly better with the ITS1 dataset in the subgenus *Astrophea* (89%) and with the ITS1 and ITS1+2 datasets in the subgenus *Passiflora* (97% and 98%, respectively), the other results showed differences among the methods.

**Table 3:** Comparison of tree-based (NJ, ML, and BI) and similarity (BM and BCM) methods performance for different ITS datasets and subgenera. The highest values of percentage of correct individuals’ identification for each subgenus and barcode region are shown in bold.

Subgenus	Barcode Region	*N* Individuals	Correct Identifications (%)				
			NJ	ML	BI	BM	BCM
*Astrophea*	ITS1	53	45.28	22.64	**88.68**	73.58	73.58
	ITS2	53	35.85	13.21	50.94	**73.58**	**73.58**
	ITS1+2	53	49.06	49.06	56.60	**73.58**	**73.58**
*Decaloba*	ITS1	314	23.25	22.58	33.76	**63.05**	61.78
	ITS2	314	21.34	15.29	29.30	**50.95**	50.31
	ITS1+2	314	31.85	33.76	38.22	**64.64**	64.01
*Deidamioides*	ITS1	101	11.88	11.88	**97.03**	96.03	95.04
	ITS2	101	29.70	2.97	28.71	**96.03**	95.04
	ITS1+2	101	28.71	27.72	**98.02**	96.03	95.04
*Passiflora *	ITS1	287	28.92	7.67	65.85	**81.53**	78.39
	ITS2	287	14.29	2.44	24.04	**54.70**	50.87
	ITS1+2	287	33.80	33.10	68.64	**81.18**	77.00

NJ, Neighbor-Joining; ML, Maximum Likelihood; BI, Bayesian inference; BM, best match; BCM, best close match.

### 2.5. Statistical Analysis

The results obtained using the BM and BCM similarity methods were significantly better than those acquired using phylogenetic trees (Table 3). The analysis performed using SPSS showed that the three ITS datasets worked equally well for the subgenera *Astrophea* and *Deidamioides*. For these subgenera, analyses conducted with the BI and BM and BCM similarity methods gave better discrimination when using these barcode loci. In the subgenus *Decaloba*, ITS1+2 performed better than other datasets and was as effective as ITS1 in the subgenus *Passiflora*. For these two subgenera, BM and BCM outperformed the tree-based methods.

## 3. Discussion

Our work includes sequences obtained from many different studies through their GenBank records. Therefore, we believe that all of them were based on correctly identified plant species, but as we are not able to identify resulting mistakes, we included at least two different sequences from different sources in our analyses. In our study, the three ITS datasets studied presented equally efficient results as potential barcodes in the subgenera *Astrophea* and *Deidamioides* as did ITS1 and ITS1+2 for the subgenus *Passiflora* and ITS1+2 for the subgenus *Decaloba*. One also must consider the steps of DNA isolation, PCR amplification, and sequencing when choosing a DNA barcode [8]; in this case, the ITS region has proved to be a suitable marker in *Passiflora* studies [36,38,43,48,49,50,51]. Neither ITS1 nor ITS2 alone were perfect to distinguish all samples in this study. *Astrophea* and *Deidamioides* subgenera presented a lower rate of variable and informative sites than *Passiflora*, while in *Decaloba* these rates are higher than in the other three subgenera. These results were expected, considering the complexity of *Passiflora* and *Decaloba* subgenera, and directly reflected on the performance of ITS1 and ITS2 as barcode marker in each subgenus. For example, *Decaloba* presented the highest rates of variable and informative sites and this is the likely reason why the rate of species discrimination is higher in this subgenus when ITS1 and ITS2 are concatenated. Even though both markers presented higher rates of species discrimination in all four *Passiflora* subgenera, ITS1 commonly presented a higher number of variable and parsimoniously informative sites for all analyzed species, although this difference was not significant. Therefore, we suggest that ITS1 itself could be the first option for DNA barcode in *Passiflora*, though ITS2 should not be discarded.

The ITS region does not always present high rates of species discrimination, and different plastid markers have already been proposed instead of ITS for several plant groups, especially *matK* (for example, *Holcoglossum*; [13]) and two combinations of plastid loci (as *Lamium*; [11]). Indeed, ITS sequences alone have been reported to be insufficient in other plants, with the combination of ITS and plastid loci being proposed [54,55]. The ITS2 region has been indicated as a DNA barcode for some plant groups [29,30,31,56]. Here, we demonstrate high rates of species discrimination based on ITS data for the *Passiflora* genus, as shown in other studies [19,23].

However, there are few studies comparing the individual performances of ITS1 and ITS2. ITS1 showed superior performance to ITS2 and several plastid regions analyzed in *Salvia* species [33], whereas [34] suggest that ITS1 should be tested first in species discrimination studies for taxonomic groups where ITS1 is known to perform better than ITS2.

In our study, the similarity-based methods generally outperformed the tree-based methods. The statistics of BM and BCM options are commonly used in plant barcoding studies to evaluate the rate of species identification [11,14,28,55]. These two similarity-based methods presented high rates of species discrimination in the *Passiflora* genus, with at least half of the species being correctly assigned. In fact, the BM and BCM results were considerably higher than those obtained for the tree-based methods NJ and ML and slightly better than those of BI, except for the subgenus *Decaloba*, for which the BI tree-based method discriminated less than 38% of species.

## 4. Experimental Section

### 4.1. Taxon Sampling

ITS1, ITS2, and ITS1+2 loci were selected as barcoding candidates. The sampling obtained (Supplementary Table S2) from GenBank included 1034 accessions from 222 *Passiflora* species representative of all four subgenera: *Astrophea* (16 spp.), *Decaloba* (134 spp.), *Deidamioides* (8 spp.), and *Passiflora* (64 spp.). On average, we analyzed four individuals per species. The number of taxa represents approximately 43% of the species richness of the *Passiflora* genus. Some of the plastid sequences tested were also obtained from GenBank (Supplementary Table S3) and included 191 accessions of 122 species for *rbcL*, 47 sequences of 22 species for *matK*, 63 accessions of 30 species for *trnH-psbA*, and 346 sequences of 185 species for the *trnL* (UAA) intron. Supplementary Table S4 includes primer sequences and references for all analyzed ITS.

### 4.2. Data Analysis

Due to its well-conserved nature, the 5.8S gene region was removed from any sequence so that the ITS1 and ITS2 regions could be analyzed separately and concatenated. The analyses were performed in each subgenus separately due to the large genetic variability observed among them. Therefore, for each marker and subgenus, sequences were automatically aligned using ClustalX [57], visually inspected, and manually adjusted using MEGA6 [58]. These software programs were also used for testing plastid sequences, but in these analyses, all four *Passiflora* subgenera were aligned together due to the reduced variability compared to the ITS region.

We evaluated the effectiveness of ITS1, ITS2, and their combination (ITS1+2) as barcodes using three different methods.

#### 4.2.1. Genetic Distance-Based Method

The barcoding gap is a measure of the effective barcode locus and is present when the minimum K2P interspecific distance is larger than the maximum intraspecific distance [5,8,11]. To estimate the barcoding gap, the TAXONDNA software [53] was used to calculate genetic distance over sequence pairs between and within species based on the K2P nucleotide substitution model. To estimate the presence of any barcoding gaps, histograms of distance *vs.* abundance were generated to evaluate whether the interspecific distances were larger than the intraspecific distances.

#### 4.2.2. DNA Sequence Similarity-Based Method

To estimate the potential of the ITS regions to identify species accurately, we measured the proportion of correct identification using a method based on a direct comparison of DNA sequences. The SpeciesIdentifier program from the TAXONDNA software package compares each sequence with all others present in the dataset and groups sequences based on their pairwise genetic distances, determining whether two sequences are likely to be conspecific. We used the “best match” (BM) and “best close match” (BCM) software functions to evaluate the proportion of successful identifications based on the K2P distance as a model. The “best match” analysis establishes the closest match for a given sequence. The identification is considered correct if both compared sequences were from the same species and incorrect if the sequences did not belong to the same species. Two or more equally good results classify the sequence as ambiguous. The “best close match” option is more stringent because it depends on 95% pairwise distance threshold calculated by the “pairwise summary” function. Results above threshold are classified as “no match”, and the remaining queries below the threshold were analyzed as in the “best match” criteria [53].

#### 4.2.3. Tree-Based Method

This analysis evaluates the proportion of monophyletic species in phylogenetic trees to assess marker discriminatory performance as a potential barcode [11,26,28]. Therefore, three different phylogenetic methods were selected for these analyses: Neighbor-Joining (NJ), maximum likelihood (ML), and Bayesian inference (BI). NJ and ML trees were constructed in MEGA using the K2P distance as a model of substitution, and running 1000 bootstrap replicates to assess the relative support for the branches. BI trees were constructed in BEAST1.8 [59] using the HKY substitution model with four gamma categories and a Yule tree prior, and 107 chain lengths were performed. The first 1000 trees were discarded as “burn in”. Species were considered correctly identified if the individuals formed a monophyletic group in the trees with a bootstrap value higher than 80% or a posterior probability greater than 0.80; these values are more stringent that those used by [26] and [60] and minimize spurious relationships due to low genetic variability in datasets. We conducted statistical analyses to evaluate the discriminatory power of each potential barcode with a two-way ANOVA test followed by a *post-hoc* Student–Newman–Keuls (SNK) test for pairwise comparisons (*p* ≤ 0.05) using the PASW Statistics18 software [61].

## 5. Conclusions

Our results show that ITS1 and ITS2 presents all the desired characteristics of a DNA barcode in *Passiflora*, such as the highest rate of discrimination and fulfillment of amplification and sequencing requirements. However, there is no ideal barcode for plants. Plastid regions were initially proposed for DNA barcoding studies [8,9] and have since been commonly used [10,54,62]. The ITS region does not always present a higher rate of species discrimination than plastid markers, though many studies indicate ITS regions as being useful for recovering high rates of correctly assigned species [24,26]. The combination of ITS and plastid loci may be chosen as the best option for some groups [25,27], and ITS2 alone is indicated as a DNA barcode for other groups [32,63]. However, ITS1 has been poorly evaluated for this purpose. Recently, it was suggested that ITS1 should be tested first as DNA barcoding when it presents better results than ITS2 for the studied taxonomic group [34]. We found that this is especially true for *Passiflora* species, and we suggest that the ITS1 region should be used as a starting point to identify species and subgenera in this highly diverse genus.

## Supplementary Materials

Supplementary materials can be found at http://www.mdpi.com/1422-0067/16/04/7289/s1.
