# Supplementary material for: Efficiency of ITS Sequences for DNA Barcoding in Passiflora (Passifloraceae)
Source: Int J Mol Sci. 2015 Apr 1;16(4):7289–303. doi: 10.3390/ijms16047289 (PMC4425017; doi:10.3390/ijms16047289)
Supplement: Supplementary file 1 [file ijms-16-07289-s001.pdf]

Supplementary Information

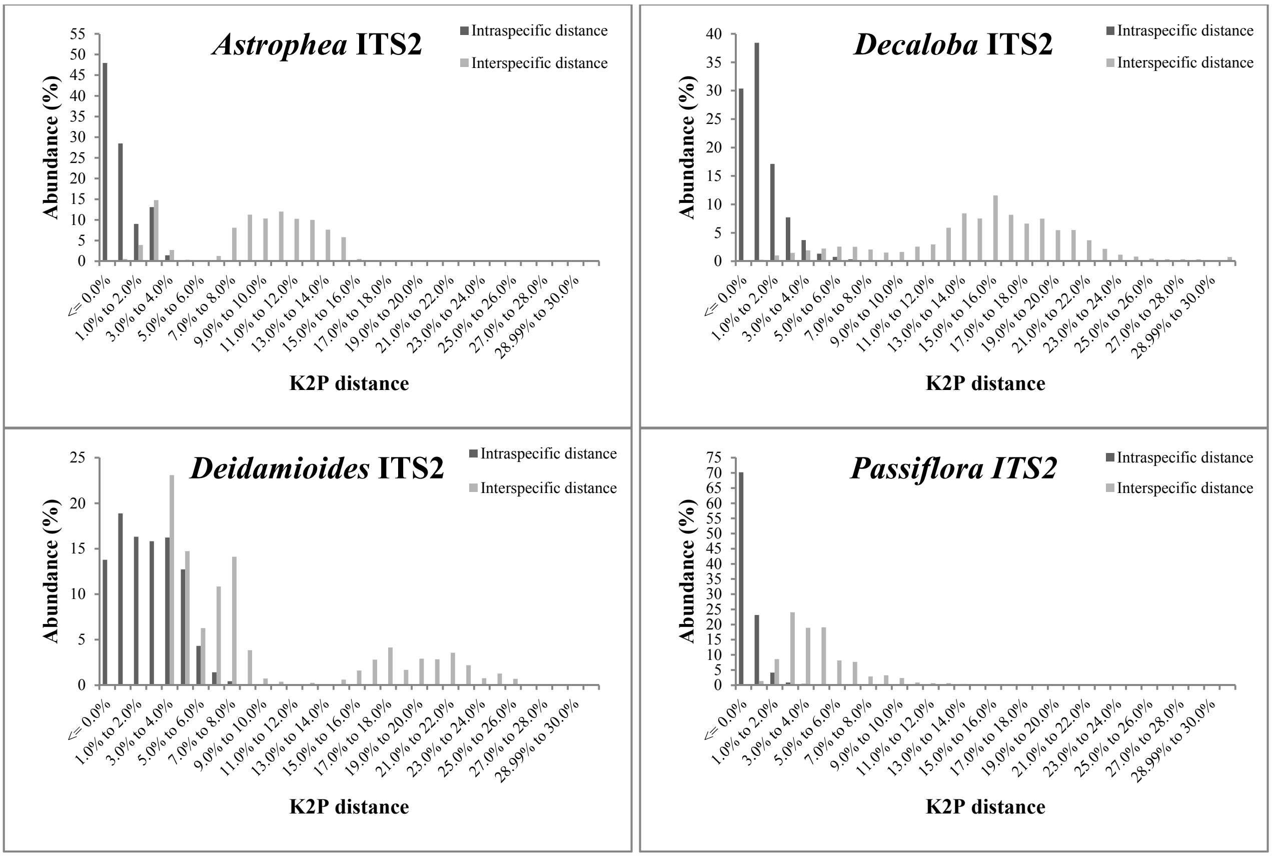

Figure S1. Relative abundance of intra- and inter-specific Kimura-2-Parameter pairwise distance considering the ITS2 dataset in subgenera *Astrophea*, *Decaloba*, *Deidamioides*, and *Passiflora*.

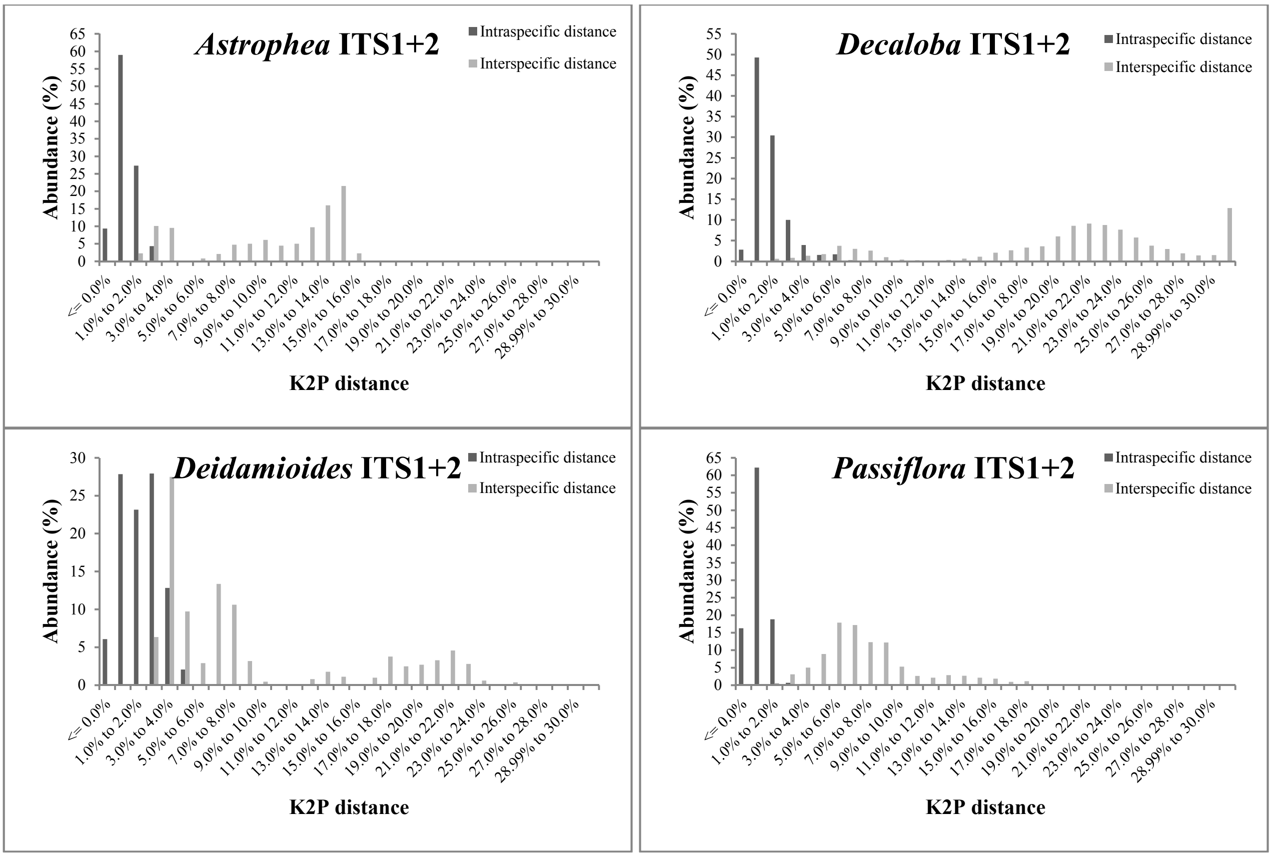

Figure S2. Relative abundance of intra- and inter-specific Kimura-2-Parameter pairwise distance considering the ITS1+2 dataset in subgenera *Astrophea*, *Decaloba*, *Deidamioides*, and *Passiflora*.

Table S1. Results for "best match" (BM) and "best close match" (BCM) analyses of TaxonDNA software for Plastid Marker *matK*, *rbcL*, *trnL-psbA*, and *trnL* intron (UAA).

Barcode Region	*N* Individuals	BM, *N* (%)			BCM, *N* (%)				
		C	A	I	C	A	I	No Match	Threshold, %
matK	47 (22 sp.)	38.29	42.55	19.14	38.29	42.55	19.14	0	14.49
rbcL	191 (122 sp.)	36.64	23.56	39.79	36.64	23.56	37.17	2.61	1.49
trnH-psbA	63 (30 sp.)	44.44	17.46	38.09	42.85	17.46	19.04	20.63	2.20
trnL intron (UAA)	346 (185 sp.)	18.78	49.13	32.08	11.84	43.35	2.60	42.19	0.0
rpoB	Four sequences available	Analysis not performed due to low availability of sequences							
rpoC1	Six sequences available								
atpF-atpH	Two sequences available								
psbK-psbI	No sequences available								

BM, best match; BCM, best close match; C, correct; A, ambiguous; I, incorrect.

Table S2. GenBank Access numbers for ITS sequences per *Passiflora* subgenera.

Astrophea ITS1		*Astrophea* ITS2	
**Species**	**GenBank Access**	**Species**	**GenBank Access**
P. amoena	KP769869 [a]	*P. amoena*	KP769917 [a]
P. arborea	JX470767 [b]	*P. arborea*	JX470767 [b]
P. candida	DQ521279 [c]	*P. candida*	DQ521279 [c]
P. ceratocarpa	KP769870 [a]	*P. ceratocarpa*	KP769918 [a]
P. citrifolia	AY210939 [d]	*P. citrifolia*	AY210920 [d]
	AY632707 [e]		AY632707 [e]
P. haematostigma	EU258395—EU258408 [f]	*P. haematostigma*	EU258395—EU258408 [f]
	EU907230—EU907234 [g]		EU907230—EU907234 [g]
	AY032835 [d]		AY032794 [d]
P. jussieu	JX470768 [b]	*P. jussieu*	JX470768 [b]
P. kawensis	KP769871 [a]	*P. kawensis*	KP769919 [a]
P. lindeniana	KP769872 [a]	*P. lindeniana*	KP769920 [a]
P. macrophylla	EU907225—EU907230 [g]	*P. macrophylla*	EU907225—EU907230 [g]
	AY210944 [d]		AY210925 [d]
	DQ458062 [h]		DQ458062 [h]
P. mansoi	AY102361 [d]	*P. mansoi*	AY102381 [d]
P. pittieri	DQ995476 [h]	*P. pittieri*	DQ995476 [h]
P. pyrrhantha	JX470771 [b]	*P. pyrrhantha*	JX470771 [b]
P. rhamnifolia	KP769873-KP769884 [a]	*P. rhamnifolia*	KP769921-KP769932 [a]
P. sphaerocarpa	JX470769 [b]	*P. sphaerocarpa*	JX470769 [b]
P. tina	JX470770 [b]	*P. tina*	JX470770 [b]

[a] Sequences from Giudicelli *et al.* (in prep); [b] Krosnick, S.E.; Porter-Utley, K.E.; MacDougal, J.M.; Jørgensen, P.M.; McDade, L.A. New insights into the evolution of *Passiflora* subgenus *Decaloba* (Passifloraceae): Phylogenetic relationships and morphological synapomorphies. *Syst. Bot.* **2013**, *38*, 692–713; [c] Hearn, D.J. Adenia (Passifloraceae) and its adaptative radiation: Phylogeny and growth form diversification. *Syst. Bot.* **2006**, *31*, 805–821; [d] Muschner, V.C.; Lorenz, A.P.; Cervi, A.C.; Bonatto, S.L.; Souza-Chies, T.T.; Salzano, F.M.; Freitas, L.B. A first molecular phylogenetic analysis of *Passiflora* (Passifloraceae). *Am. J. Bot.* **2003**, *90*, 1229–1238; [e] Krosnick, S.E.; Freudenstein, J.V. Monophyly and floral character homology of old world *Passiflora* (Subgenus *Decaloba*: Supersection *Disemma*). *Syst. Bot.* **2005**, 30, 139–152; [f] Mäder, G.; Zamberlan, P.M.; Fagundes, N.J.R.; Magnus, T.; Salzano, F.M.; Bonatto, S.L.; Freitas, L.B. The use and limits of ITS data in the analysis of intraspecific variation in *Passiflora* L. (Passifloraceae). *Genet. Mol. Biol.* **2010**, *33*, 99–108; [g] Mäder, G.; Magnus, T.; Lorenz-Lemke, A.P.; *et al.* ITS subgenera and intraspecific variability in Brazilian *Passiflora*: Understandin molecular evolution. Unpublished; [h] Krosnick, S.E.; Ford, A.; Freudenstein, J.V. Resolving the phylogenetic position of *Hollrungia* and *Tetrapathaea*: The end of two monotypic genera in Passifloraceae. Unpublished.

Table S2. *Cont.*

Decaloba ITS1		*Decaloba* ITS2	
**Species**	**GenBank Access**	**Species**	**GenBank Access**
P. adenopoda	AY632702 [a]	*P. adenopoda*	AY632702 [a]
P. allantophylla	DQ458069 [b]	*P. allantophylla*	DQ458069 [b]
P. altebilobata	DQ458078 [b]	*P. altebilobata*	DQ458078 [b]
P. anadenia	JX470833 [c]	*P. anadenia*	JX470833 [c]
P. apetala	JX470822 [c]	*P. apetala*	JX470822 [c]
P. apoda	JX470779 [c]	*P. apoda*	JX470779 [c]
P. aurantia	DQ521280 [d]	*P. aurantia*	DQ521280 [d]
	AY632704 [a]		AY632704 [a]
P. auriculata	AF454804 [e]	*P. auriculata*	AF454804 [e]
	DQ284532 [f]		DQ284532 [f]
P. berteroana	JX470780 [c]	*P. berteroana*	JX470780 [c]
P. bicornis	JX470836 [c]	*P. bicornis*	JX470836 [c]
P. bicrura	JX470834 [c]	*P. bicrura*	JX470834 [c]
P. biflora	DQ521281 [d]	*P. biflora*	DQ521281 [d]
	AF454805 [e]		AF454805 [e]
	AY632705 [a]		AY632705 [a]
	JX470837 [c]		JX470837 [c]
P. boendery	JX470823 [c]	*P. boendery*	JX470823 [c]
P. bryonioides	JX470796 [c]	*P. bryonioides*	JX470796 [c]
P. calcicola	JX470813 [c]	*P. calcicola*	JX470813 [c]
P. capsularis	EU258327—EU258351 [g]	*P. capsularis*	EU258327—EU258351 [g]
	EU907235—EU907250 [h]		EU907235—EU907250 [h]
	AY032837 [i]		AY032796 [i]
	JX470806 [c]		JX470806 [c]
P. chelidonea	JX470838 [c]	*P. chelidonea*	JX470838 [c]
P. chrysosepala	JX470839 [c]	*P. chrysosepala*	JX470839 [c]
P. cinnabarina	AY632706 [a]	*P. cinnabarina*	AY632706 [a]
P. citrina	DQ458083 [b]	*P. citrina*	DQ458083 [b]
	JX463165 [c]		JX463165 [c]
P. cobanensis	JX470807 [c]	*P. cobanensis*	JX470807 [c]
P. cochinchinensis	DQ458080 [b]	*P. cochinchinensis*	DQ458080 [b]
	DQ087422		DQ087422
	AY632714 [a]		AY632714 [a]
P. colimensis	JX470797 [c]	*P. colimensis*	JX470797 [c]
P. complanata	JX470827 [c]	*P. complanata*	JX470827 [c]
P. coriacea	AF454807 [e]	*P. coriacea*	AF454807 [e]
	AY210940 [i]		AY210921 [i]
	DQ238786 [f]		DQ238786 [f]
	JX463147 [c]		JX463147 [c]
	JX470790 [c]		JX470790 [c]
P. cubensis	JX470814 [c]	*P. cubensis*	JX470814 [c]
P. cuneata	JX470840 [c]	*P. cuneata*	JX470840 [c]
P. cupiformis	AY632708 [a]	*P. cupiformis*	AY632708 [a]

Table S2. *Cont.*

Decaloba ITS1		*Decaloba* ITS2	
**Species**	**GenBank Access**	**Species**	**GenBank Access**
P. cupraea	AY210941 [i]	*P. cupraea*	AY210922 [i]
	JX470815 [c]		JX470815 [c]
P. dolichocarpa	JX470798 [c]	*P. dolichocarpa*	JX470798 [c]
P. eberhardtii	DQ458073 [b]	*P. eberhardtii*	DQ458073 [b]
	JX470778 [c]		JX470778 [c]
P. ekmanii	JX470835 [c]	*P. ekmanii*	JX470835 [c]
P. escobariana	JX470808 [c]	*P. escobariana*	JX470808 [c]
P. exsudans	JX470799 [c]	*P. exsudans*	JX470799 [c]
P. geminiflora	DQ458075—DQ458076 [b]	*P. geminiflora*	DQ458075—DQ458076 [b]
P. gilbertiana	JX470824 [c]	*P. gilbertiana*	JX470824 [c]
P. gracilis	JX470800 [c]	*P. gracilis*	JX470800 [c]
P. guatemalensis	DQ087419 [f]	*P. guatemalensis*	DQ087419 [f]
P. hahnii	JX470777 [c]	*P. hahnii*	JX470777 [c]
P. helleri	AY210942 [i]	*P. helleri*	AY210923 [i]
	DQ458082 [b]		DQ458082 [b]
P. henryi	AY632710 [a]	*P. henryi*	AY632710 [a]
P. herbertiana	AY632711 [a]	*P. herbertiana*	AY632711 [a]
P. hirtiflora	JX470841 [c]	*P. hirtiflora*	JX470841 [c]
P. hollrungii	DQ458081 [b]	*P. hollrungii*	DQ458081 [b]
P. holosericea	DQ087417 [f]	*P. holosericea*	DQ087417 [f]
	JX470781 [c]		JX470781 [c]
P. ichthyura	JX470842 [c]	*P. ichthyura*	JX470842 [c]
P. ilamo	JX470825 [c]	*P. ilamo*	JX470825 [c]
P. inca	JX463163 [c]	*P. inca*	JX463163 [c]
P. indecora	JX470843 [c]	*P. indecora*	JX470843 [c]
P. intricata	JX470844 [c]	*P. intricata*	JX470844 [c]
P. jianfengensis	DQ458077 [b]	*P. jianfengensis*	DQ458077 [b]
P. jugorum	AY632712 [a]	*P. jugorum*	AY632712 [a]
P. juliana	JX463152—JX463154 [c]	*P. juliana*	JX463152—JX463154 [c]
	JX470791 [c]		JX470791 [c]
P. karwinskii	JX470801 [c]	*P. karwinskii*	JX470801 [c]
P. kwangtungensis	KF207865 [j]	*P. kwangtungensis*	KF207865 [j]
P. lancearia	JX470845 [c]	*P. lancearia*	JX470845 [c]
P. lancetillensis	AY210943 [i]	*P. lancetillensis*	AY210924 [i]
P. lancifolia	JX463158 [c]	*P. lancifolia*	JX463158 [c]
	JX470792 [c]		JX470792 [c]
P. leptoclada	JX470846 [c]	*P. leptoclada*	JX470846 [c]
P. leschenaultii	DQ458079 [b]	*P. leschenaultii*	DQ458079 [b]
P. litoralis	JX463107 [c]	*P. litoralis*	JX463107 [c]
	JX463109 [c]		JX463109 [c]
	JX463112—JX463118 [c]		JX463112—JX463118 [c]
	JX463123—JX463126 [c]		JX463123—JX463126 [c]
	JX463133—JX463134 [c]		JX463133—JX463134 [c]

Table S2. *Cont.*

***Decaloba* ITS1**		***Decaloba* ITS2**	
**Species**	**GenBank Access**	**Species**	**GenBank Access**
P. lobata	AF454808 [e]	*P. lobata*	AF454808 [e]
	JX463164 [c]		JX463164 [c]
	JX470802 [c]		JX470802 [c]
P. lobbii	JX463162 [c]	*P. lobbii*	JX463162 [c]
P. lobbii subsp. *ayacuchoensis*	JX470782 [c]	*P. lobbii* subsp. *ayacuchoensis*	JX470782 [c]
P. lutea	DQ006022 [k]	*P. lutea*	DQ006022 [k]
P. maestrensis	JX470816 [c]	*P. maestrensis*	JX470816 [c]
P. mcvaughiana	JX463148—JX463149 [c]	*P. mcvaughiana*	JX463148—JX463149 [c]
P. membranacea	AY632701 [a]	*P. membranacea*	AY632701 [a]
P. mexicana	AY632713 [a]	*P. mexicana*	AY632713 [a]
P. micropetala	KP769908 [l]	*P. micropetala*	KP769956 [l]
	JX470847 [c]		JX470847 [c]
P. microstipula	DQ458066 [m]	*P. microstipula*	DQ458066 [m]
P. misera	EU258409—EU258413 [g]	*P. misera*	EU258409—EU258413 [g]
	AY032838 [i]		AY032797 [i]
	JX470848 [c]		JX470848 [c]
P. moluccana var. *glaberrima*	DQ284536 [f]	*P. moluccana* var. *glaberrima*	DQ284536 [f]
P. monadelpha	DQ087418 [f]	*P. monadelpha*	DQ087418 [f]
	JX470783 [c]		JX470783 [c]
P. morifolia	EU258323—EU258324 [g]	*P. morifolia*	EU258323—EU258324 [g]
	AY032842 [i]		AY032801 [i]
	DQ284533 [f]		DQ284533 [f]
P. multiflora	AY210945 [i]	*P. multiflora*	AY210926 [i]
	AY632715		AY632715
P. munchiquensis	JX470784 [c]	*P. munchiquensis*	JX470784 [c]
P. murucuja	AY648559 [n]	*P. murucuja*	AY648559 [n]
	JX470817 [c]		JX470817 [c]
P. oblongata	JX470818 [c]	*P. oblongata*	JX470818 [c]
P. obtusifolia	JX463150—JX463151 [c]	*P. obtusifolia*	JX463150—JX463151 [c]
	JX470793 [c]		JX470793 [c]
P. occidentalis	JX470849 [c]	*P. occidentalis*	JX470849 [c]
P. orbiculata	JX470819 [c]	*P. orbiculata*	JX470819 [c]
P. organensis	EU258414—EU258426 [g]	*P. organensis*	EU258414—EU258426 [g]
	AY032839 [i]		AY032798 [i]
P. ornithoura	JX470826 [c]	*P. ornithoura*	JX470826 [c]
P. pallida	DQ458084 [b]	*P. pallida*	DQ458084 [b]
	JX463127—JX463132 [c]		JX463127—JX463132 [c]
	JX463135—JX463142 [c]		JX463135—JX463142 [c]
P. papilio	DQ458074 [b]	*P. papilio*	DQ458074 [b]
P. pardifolia	JX470850 [c]	*P. pardifolia*	JX470850 [c]
P. pavonis	JX470831 [c]	*P. pavonis*	JX470831 [c]

Table S2. *Cont.*

***Decaloba* ITS1**		***Decaloba* ITS2**	
**Species**	**GenBank Access**	**Species**	**GenBank Access**
P. pedicellaris	JX470776 [c]	*P. pedicellaris*	JX470776 [c]
P. pendens	JX470803 [c]	*P. pendens*	JX470803 [c]
P. penduliflora	KP769909 [l]	*P. penduliflora*	KP769957 [l]
	JX463166 [c]		JX463166 [c]
	JX470820 [c]		JX470820 [c]
P. perakensis	DQ087423 [f]	*P. perakensis*	DQ087423 [f]
P. perfoliata	JX463167 [c]	*P. perfoliata*	JX463167 [c]
	JX470821 [c]		JX470821 [c]
P. pilosa	JX470804 [c]	*P. pilosa*	JX470804 [c]
P. podlechii	KP769910 [l]	*P. podlechii*	KP769958 [l]
	JX463161 [c]		JX463161 [c]
P. pohlii	EU258325 [g]	*P. pohlii*	EU258325 [g]
	AY032840 [i]		AY032799 [i]
P. punctata	AY210946 [i]	*P. punctata*	AY210927 [i]
	JX470851 [c]		JX470851 [c]
P. pusilla	JX470809 [c]	*P. pusilla*	JX470809 [c]
P. rovirosae	KP769911 [l]	*P. rovirosae*	KP769959 [l]
	JX470810 [c]		JX470810 [c]
P. rubra	AY032836 [i]	*P. rubra*	AY032795 [i]
	AY632716 [a]		AY632716 [a]
	JX470811 [c]		JX470811 [c]
P. rufa	AY210948 [i]	*P. rufa*	AY210929 [i]
	JX470789 [c]		JX470789 [c]
P. rugosissima	JX470828 [c]	*P. rugosissima*	JX470828 [c]
P. sagasteguii	JX470785 [c]	*P. sagasteguii*	JX470785 [c]
P. sandrae	JX470852 [c]	*P. sandrae*	JX470852 [c]
P. sanguinolenta	KP769912 [l]	*P. sanguinolenta*	KP769960 [l]
	JX470812 [c]		JX470812 [c]
P. sexflora	AY210949 [i]	*P. sexflora*	AY210930 [i]
	JX463168[c]		JX463168 [c]
	JX470829—JX470830 [c]		JX470829—JX470830 [c]
P. sexocellata	JX463143—JX463146 [c]	*P. sexocellata*	JX463143—JX463146 [c]
P. siamica	DQ458212—DQ458216 [b]	*P. siamica*	DQ458212—DQ458216 [b]
	DQ087424 [f]		DQ087424 [f]
	AY632717 [a]		AY632717 [a]
P. sicyoides	JX470805 [c]	*P. sicyoides*	JX470805 [c]
P. sodiroi	JX470786 [c]	*P. sodiroi*	JX470786 [c]
P. solomonii	JX470787 [c]	*P. solomonii*	JX470787 [c]
P. suberosa	AY032841 [i]	*P. suberosa*	AY032800 [i]
	AF454806 [e]		AF454806 [e]
	AY632718 [a]		AY632718 [a]
P. suberosa var. *suberosa*	JX463108 [c]	*P. suberosa* var. *suberosa*	JX463108 [c]

Table S2. *Cont.*

***Decaloba* ITS1**		***Decaloba* ITS2**	
**Species**	**GenBank Access**	**Species**	**GenBank Access**
	JX463110—JX463111 [c]		JX463110—JX463111 [c]
	JX463119—JX463122 [c]		JX463119—JX463122 [c]
P. tacanensis	JX470794 [c]	*P. tacanensis*	JX470794 [c]
P. talamancensis	AF454809 [e]	*P. talamancensis*	AF454809 [e]
P. tatei	JX470853 [c]	*P. tatei*	JX470853 [c]
P. telesiphe	JX470854 [c]	*P. telesiphe*	JX470854 [c]
P. tenella	JX470832 [c]	*P. tenella*	JX470832 [c]
P. tenuiloba	AY632719 [a]	*P. tenuiloba*	AY632719 [a]
	JX463159—JX463160 [c]		JX463159—JX463160 [c]
P. tonkinensis	DQ087425 [f]	*P. tonkinensis*	DQ087425 [f]
P. transversalis	KP769913 [l]	*P. transversalis*	KP769961 [l]
P. tricuspis	EU258455—EU258460 [g]	*P. tricuspis*	EU258455—EU258460 [g]
	AY102348 [i]		AY102368 [i]
	JX470855 [c]		JX470855 [c]
P. trifasciata	KP769885 [l]	*P. trifasciata*	KP769933 [l]
P. truncata	AY102354 [i]	*P. truncata*	AY102374 [i]
	JX470788[c]		JX470788 [c]
P. tuberosa	JX470856 [c]	*P. tuberosa*	JX470856 [c]
P. tulae	AY102352 [i]	*P. tulae*	AY102372 [i]
P. urnifolia	EU258461—EU258465 [g]	*P. urnifolia*	EU258461—EU258465 [g]
	JX470857 [c]		JX470857 [c]
P. vespertilio	KP769916 [l]	*P. vespertilio*	KP769964 [l]
	JX470858 [c]		JX470858 [c]

Table S2. *Cont.*

Decaloba ITS1		*Decaloba* ITS2	
**Species**	**GenBank Access**	**Species**	**GenBank Access**
P. viridescens	JX470859 [c]	*P. viridescens*	JX470859 [c]
P. viridiflora	JX463155—JX463157 [c]	*P. viridiflora*	JX463155—JX463157 [c]
P. wilsonii	DQ458072 [b]	*P. wilsonii*	DQ458072 [b]
	DQ087426		DQ087426
P. xiizkodz	AY210950 [i]	*P. xiizkodz*	AY210931 [i]
	DQ238786 [o]		DQ238786 [o]
	JX463102—JX463106 [c]		JX463102—JX463106 [c]
	JX470795 [c]		JX470795 [c]
P. xiizkodz subsp. i*tzensis*	JX463101 [c]	*P. xiizkodz* subsp. *itzensis*	JX463101 [c]
P. xishuangbannaensis	DQ458071 [b]	*P. xishuangbannaensis*	DQ458071 [b]

[a] Krosnick, S.E.; Freudenstein, J.V. Monophyly and floral character homology of old world *Passiflora* (Subgenus *Decaloba*: Supersection *Disemma*). *Syst. Bot.* **2005**, *30*, 139–152; [b] Krosnick, S.E.; Freudenstein, J.V. Phylogenetic relationships among the Old World species of *Passiflora* L. (Subgenus *Decaloba*: Supersection *Disemma*). Unpublished; [c] Krosnick, S.E.; Porter-Utley, K.E.; MacDougal, J.M.; Jørgensen, P.M.; McDade, L.A. New insights into the evolution of *Passiflora* subgenus *Decaloba* (Passifloraceae): phylogenetic relationships and morphological synapomorphies. *Syst. Bot.* **2013**, *38*, 692–713; [d] Hearn, D.J. *Adenia* (Passifloraceae) and its adaptative radiation: Phylogeny and growth form diversification. *Syst. Bot.* **2006**, *31*, 805–821; [e] Ossowski, A.M.; Hunter, F.F. Coevolution of *Heliconius* spp. and *Passiflora* spp.: A phylogenetic comparison. Unpublished; [f] Krosnick, S.E.; Freudenstein, J.V. Patterns of anomalous floral development in the Asian *Passiflora* (subgenus *Decaloba*: supersection *Disemma*). *Am. J. Bot.* **2006**, *93*, 620–636; [g] Mäder, G.; Zamberlan, P.M.; Fagundes, N.J.R.; Magnus, T.; Salzano, F.M.; Bonatto, S.L.; Freitas, L.B. The use and limits of ITS data in the analysis of intraspecific variation in *Passiflora* L. (Passifloraceae). *Genet. Mol. Biol.* **2010**, *33*, 99–108; [h] Mäder, G.; Magnus, T.; Lorenz-Lemke, A.P.; *et al.* ITS subgenera and intraspecific variability in Brazilian *Passiflora*: Understandin molecular evolution. Unpublished; [i] Muschner, V.C.; Lorenz, A.P.; Cervi, A.C.; Bonatto, S.L.; Souza-Chies, T.T.; Salzano, F.M.; Freitas, L.B. A first molecular phylogenetic analysis of Passiflora (Passifloraceae). *Am. J. Bot.* **2003**, *90*, 1229–1238; [j] Krosnick, S.E.; Xun-Lin, Y.; Deng, Y. The rediscovery of *Passiflora kwangtungensis* Merr. (subgenus *Decaloba* supersection *Disemma*): A critically endangered Chinese endemic. *PhytoKeys* **2013**, *23*, 55–74; [k] Kress, W.J.; Wurdack, K.J.; Zimmer, E.A.; Weigt, L.A.; Janzen, D.H. Use of DNA barcodes to identify flowering plants. *Proc. Natl. Acad. Sci. USA* **2005**, *102*, 8369–8374; [l] Sequences from Giudicelli *et al.* (in prep); [m] Krosnick, S.E.; Ford, A.; Freudenstein, J.V. Resolving the phylogenetic position of *Hollrungia* and *Tetrapathaea*: The end of two monotypic genera in Passifloraceae. Unpublished; [n] Kay, E.E. Floral Evolutionary Ecology of Passiflora: subgenera Murucuia, Pseudomurucuja and Astephia. Unpublished; [o] Muschner, V.C.; Lorenz-Lemke, A.P.; Vecchia, M.; Bonatto, S.L.; Salzano, F.M.; Freitas, L.B. Differential organellar inheritance in *Passiflora* (Passifloraceae) subgenera. Unpublished.

Table S2. *Cont.*

***Deidamioides* ITS1**		***Deidamioides* ITS2**	
**Species**	**GenBank Access**	**Species**	**GenBank Access**
P. arbelaezii	DQ521278 [a]	*P. arbelaezii*	DQ521278 [a]
	AY632703 [b]		AY632703 [b]
P. cirrhiflora	DQ458063 [c]	*P. cirrhiflora*	DQ458063 [c]
P. contracta	KF196619—KF196691 [d]	*P. contracta*	KF196619—KF196691 [d]
P. deidamioides	EU907257—EU907265 [e]	*P. deidamioides*	EU907257—EU907265 [e]
P. discophora	DQ458061 [c]	*P. discophora*	DQ458061 [c]
	JX470772 [f]		JX470772 [f]
P. gracillima	JX470773 [f]	*P. gracillima*	JX470773 [f]
P. obovata	DQ458064 [c]	*P. obovata*	DQ458064 [c]
P. ovalis	KF196601—KF196618 [d]	*P. ovalis*	KF196601—KF196618 [d]

[a] Hearn, D.J. *Adenia* (Passifloraceae) and its adaptative radiation: Phylogeny and growth form diversification. *Syst. Bot.* **2006**, *31*, 805–821; [b] Krosnick, S.E.; Freudenstein, J.V. Monophyly and floral character homology of old world *Passiflora* (Subgenus *Decaloba*: Supersection *Disemma*). *Syst. Bot.* **2005**, *30*, 139–152; [c] Krosnick, S.E.; Ford, A.; Freudenstein, J.V. Resolving the phylogenetic position of *Hollrungia* and *Tetrapathaea*: The end of two monotypic genera in Passifloraceae. Unpublished; [d] Cazé, A.L.R.; Mäder, G.; Bonatto, S.L.; Freitas, L.B. A molecular systematic analysis of *Passiflora ovalis* and *Passiflora contracta* (Passifloraceae). *Phytotaxa* **2013**, *132*, 39–46; [e] Mäder, G.; Magnus, T.; Lorenz-Lemke, A.P.; *et al.* ITS subgenera and intraspecific variability in Brazilian *Passiflora*: Understandin molecular evolution. Unpublished; [f] Krosnick, S.E.; Porter-Utley, K.E.; MacDougal, J.M.; Jørgensen, P.M.; McDade, L.A. New insights into the evolution of *Passiflora* subgenus *Decaloba* (Passifloraceae): Phylogenetic relationships and morphological synapomorphies. *Syst. Bot.* **2013**, *38*, 692–713.

Table S2. *Cont.*

Passiflora ITS1		*Passiflora* ITS2	
**Species**	**GenBank Access**	**Species**	**GenBank Access**
P. actinia	AY032832 [a]	*P. actinia*	AY032791 [a]
	AY542629—AY542644 [b]		AY219264—AY219279 [b]
	AY219240—AY219255 [b]		AY542658—AY542673 [b]
P. acuminata	KP769886 [c]	*P. acuminata*	KP769934 [c]
P. alata	AY032826 [a]	*P. alata*	AY032785 [a]
	AY858145—AY858229 [d]		AY858263—AY858347 [d]
	AF454800 [e]		AF454800 [e]
P. ambigua	AF454801 [e]	*P. ambigua*	AF454801 [e]
P. amethystina	EU258307—EU258309 [f]	*P. amethystina*	EU258307—EU258309 [f]
	AY102347 [a]		AY102367 [a]
P. ampullacea	AY632720 [g]	*P. ampullacea*	AY632720 [g]
P. caerulea	EU258310—EU258316 [f]	*P. caerule\a*	EU258310—EU258316 [f]
	AY032824 [a]		AY032782 [a]
	AF454802 [e]		AF454802 [e]
P. campanulata	AY032829 [a]	*P. campanulata*	AY032788 [a]
P. cerasina	KP769887 [c]	*P. cerasina*	KP769935 [c]
P. chrysophylla	KP769906 [c]	*P. chrysophylla*	KP769954 [c]
P. cincinnata	EU258353—EU258358 [f]	*P. cincinnata*	EU258353—EU258358 [f]
	DQ344629 [h]		DQ344629 [h]
	AY102363 [a]		AY102383 [a]

Table S2. *Cont.*

Passiflora ITS1		*Passiflora* ITS2	
**Species**	**GenBank Access**	**Species**	**GenBank Access**
P. coccinea	KP769888 [c]	*P. coccinea*	KP769936 [c]
P. edmundoi	EU258370 [f]	*P. edmundoi*	EU258370 [f]
	EU258373—EU258374 [f]		EU258373—EU258374 [f]
	AY102351 [a]		AY102371 [a]
P. edulis	EU258375—EU258384 [f]	*P. edulis*	EU258375–EU258384 [f]
	AY032831 [a]		AY032790 [a]
	JX470774 [i]		JX470774 [i]
	AF454803 [e]		AF454803 [e]
P. eichleriana	EU258317—EU258319 [f]	*P. eichleriana*	EU258317—EU258319 [f]
	AY102346 [a]		AY102366 [a]
P. elegans	AY032833 [a]	*P. elegans*	AY032792 [a]
	AY542645—AY542657 [b]		AY219280—AY219286 [b]
	AY219256—AY219262 [b]		AY542674—AY542686 [b]
P. foetida	DQ521376 [j]	*P. foetida*	DQ521376 [j]
	EU258385—EU258390 [f]		EU258385—EU258390 [f]
	EU258393—EU258394 [f]		EU258393—EU258394 [f]
	AY032834 [a]		AY032793 [a]
	DQ238783 [h]		DQ238783 [h]
	DQ458053 [k]		DQ458053 [k]
	DQ499117 [l]		DQ499117 [l]
	JQ723359 [m]		JQ723359 [m]
P. gabrielliana	AY210953 [a]	*P. gabrielliana*	AY210934 [a]
P. galbana	AY032843 [a]	*P. galbana*	AY032784 [a]
P. garckei	AY210952 [a]	*P. garckei*	AY210933 [a]
P. glandulosa	KP769907 [c]	*P. glandulosa*	KP769955 [c]
P. hatsbachii	KP769889 [c]	*P. hatsbachii*	KP769937 [c]
P. incarnata	DQ344630 [h]	*P. incarnata*	DQ344630 [h]
P. ishnoclada	KP769890 [c]	*P. ishnoclada*	KP769938 [c]
P. jervensis	KP769891 [c]	*P. jervensis*	KP769939 [c]
P. jilekii	EU258320—EU258321 [f]	*P. jilekii*	EU258320—EU258321 [f]
	AY102360 [a]		AY102380 [a]
P. kermesina	AY032825 [a]	*P. kermesina*	AY032783 [a]
P. laurifolia	KP769892 [c]	*P. laurifolia*	KP769940 [c]
P. loefgrenii	KP769893 [c]	*P. loefgrenii*	KP769941 [c]
P. luetzelburi	KP769894 [c]	*P. luetzelburi*	KP769942 [c]
P. maliformis	AY210956 [a]	*P. maliformis*	AY210937 [a]
P. mathewsii	KP769895 [c]	*P. mathewsii*	KP769943 [c]
P. mendoncaei	AY102358 [a]	*P. mendoncaei*	AY102378 [a]
P. menispermifolia	AF454795 [e]	*P. menispermifolia*	AF454795 [e]
P. miersii	EU258322 [f]	*P. miersii*	EU258322 [f]
	EU907266—EU907269 [n]		EU907266—EU907269 [n]
	AY102350 [a]		AY102370 [a]

Table S2. *Cont.*

***Passiflora* ITS1**		***Passiflora* ITS2**	
**Species**	**GenBank Access**	**Species**	**GenBank Access**
P. mixta	KP769896 [c]	*P. mixta*	KP769944 [c]
P. mucronata	AY210951 [a]	*P. mucronata*	AY210932 [a]
P. mucugensis	KP769897 [c]	*P. mucugensis*	KP769945 [c]
P. nitida	KP769898 [c]	*P. nitida*	KP769946 [c]
P. odontophylla	KP769899 [c]	*P. odontophylla*	KP769947 [c]
P. oerstedii	AF454797 [e]	*P. oerstedii*	AF454797 [e]
P. palmeri	DQ238784 [h]	*P. palmeri*	DQ238784 [h]
P. pilosicorona	KP769900 [c]	*P. pilosicorona*	KP769948 [c]
P. platyloba	AF454798 [e]	*P. platyloba*	AF454798 [e]
P. quadrangularis	AY032827 [a]	*P. quadrangularis*	AY032786 [a]
	AY636107 [g]		AY636107 [g]
	AF454799 [e]		AF454799 [e]
P. racemosa	KP769901 [c]	*P. racemosa*	KP769949 [c]
P. recurva	AY102349 [a]	*P. recurva*	AY102369 [a]
P. reflexiflora	AY210947 [a]	*P. reflexiflora*	AY210928 [a]
P. serratifolia	AY210954 [a]	*P. serratifolia*	AY210935 [a]
P. serratodigitata	AY636108 [g]	*P. serratodigitata*	AY636108 [g]
	AY210957 [a]		AY210938 [a]
P. setacea	AY102356 [a]	*P. setacea*	AY102376 [a]
P. setulosa	AY032828 [a]	*P. setulosa*	AY032787 [a]
P. sidiifolia	EU258435—EU258445 [f]	*P. sidiifolia*	EU258435—EU258445 [f]
	AY102353 [a]		AY102373 [a]
P. speciosa	AY102362 [a]	*P. speciosa*	AY102382 [a]
P. sprucei	KP769902 [c]	*P. sprucei*	KP769950 [c]
P. tenuifila	EU258446—EU258454 [f]	*P. tenuifila*	EU258446—EU258454 [f]
P. trifoliata	KP769903 [c]	*P. trifoliata*	KP769951 [c]
P. trintae	KP769914 [c]	*P. trintae*	KP769962 [c]

Table S2. *Cont.*

Passiflora ITS1		*Passiflora* ITS2	
**Species**	**GenBank Access**	**Species**	**GenBank Access**
P. tripartita	KP769904 [c]	*P. tripartita*	KP769952 [c]
P. trisecta	KP769905 [c]	*P. trisecta*	KP769953 [c]
P. umbilicata	KP769915 [c]	*P. umbilicata*	KP769963 [c]
P. urubiciensis	EU258326 [f]	*P. urubiciensis*	EU258326 [f]
	AY102355 [a]		AY102375 [a]
P. villosa	EU258391—EU258392 [f]	*P. villosa*	EU258391—EU258392 [f]
	EU258466—EU258469 [f]		EU258466—EU258469 [f]
	AY102357 [a]		AY102377 [a]
P. vitifolia	AF454796 [e]	*P. vitifolia*	AF454796 [e]

[a] Muschner, V.C.; Lorenz, A.P.; Cervi, A.C.; Bonatto, S.L.; Souza-Chies, T.T.; Salzano, F.M.; Freitas, L.B. A first molecular phylogenetic analysis of Passiflora (Passifloraceae). *Am. J. Bot.* **2003**, *90*, 1229–1238; [b] Lorenz-Lemke, A.P.; Muschner, V.C.; Bonatto, S.L.; Cervi, A.C.; Salzano, F.M.; Freitas, L.B. Phylogeographic inferences concerning evolution of Brazilian *Passiflora actinia* and *P. elegans* (Passifloraceae) based on ITS (nrDNA) variation. *Ann. Bot.* **2005**, *95*, 799–806; [c] Sequences from Giudicelli *et al.* (in prep); [d] Koehler-Santos, P.; Lorenz-Lemke, A.P.; Muschner, V.C.; Salzano, F.M.; Freitas, L.B. Evolutionary implications of the intrapopulation diversity of *Passiflora alata*. Unpublished; [e] Ossowski, A.M.; Hunter, F.F. Coevolution of *Heliconius* spp. and *Passiflora* spp.: A phylogenetic comparison. Unpublished; [f] Mäder, G.; Zamberlan, P.M.; Fagundes, N.J.R.; Magnus, T.; Salzano, F.M.; Bonatto, S.L.; Freitas, L.B. The use and limits of ITS data in the analysis of intraspecific variation in *Passiflora* L. (Passifloraceae). *Genet. Mol. Biol.* **2010**, *33*, 99–108; [g] Krosnick, S.E.; Freudenstein, J.V. Monophyly and floral character homology of old world *Passiflora* (Subgenus *Decaloba*: Supersection *Disemma*). *Syst. Bot.* **2005**, *30*, 139–152; [h] Muschner, V.C.; Lorenz-Lemke, A.P.; Vecchia, M.; Bonatto, S.L.; Salzano, F.M.; Freitas, L.B. Differential organellar inheritance in *Passiflora* (Passifloraceae) subgenera. Unpublished; [i] Krosnick, S.E.; Porter-Utley, K.E.; MacDougal, J.M.; Jørgensen, P.M.; McDade, L.A. New insights into the evolution of *Passiflora* subgenus *Decaloba* (Passifloraceae): phylogenetic relationships and morphological synapomorphies. *Syst. Bot.* **2012**, *38*, 692–713; [j] Hearn, D.J. *Adenia* (Passifloraceae) and its adaptative radiation: Phylogeny and growth form diversification. *Syst. Bot.* **2006**, *31*, 805–821; [k] Krosnick, S.E.; Ford, A.; Freudenstein, J.V. Resolving the phylogenetic position of *Hollrungia* and *Tetrapathaea*: The end of two monotypic genera in Passifloraceae. Unpublished; [l] Wright, S.; Keeling, J.; Gillman, L. The road from santa Rosalia: A faster tempo of evolution on tropical climes. *Proc. Natl. Acad. Sci. USA* **2006**, *103*, 7718–7722; [m] Thulin, M.; Razafimandimbison, S.G.; Chafe, P.; Heidari, N.; Kool, A.; Shore, J.S. Phyloheny of the Turneracea clade (Passifloraceae): Trans-Atlantic disjunctions and two new genera in Africa. *Taxon* **2012**, *61*, 308–323; [n] Mäder, G.; Magnus, T.; Lorenz-Lemke, A.P.; *et al.* ITS subgenera and intraspecific variability in Brazilian *Passiflora*: Understandin molecular evolution. Unpublished.

Table S3. GenBank access numbers for Plastid Markers *matK*, *rbcL*, *trnL-psbA*, and *trnL* intron (UAA).

Plastid Marker	Species	GenBank Access
matK	*P. adenopoda*	AY271608 [1]
	P. ambigua	JQ588571 [2]
	P. aurantioides	AB536631 [3]
	P. bicornis	JQ588572–JQ588574 [2]
	P. biflora	AY271610 [1]
		EU017067 [4]
		GU135122 [5]
		JQ588575–JQ588578 [2]
	P. caerulea	HM850927 [6]
	P. capsularis	AY271611 [1]
	P. cf. *wilsonii*	HG004937 [7]
	P. ciliata	JX661956 [8]
	P. coccinea	EF135577 [9]
	P. coriacea	AY271609 [1]
	P. costaricensis	JQ588579–JQ588580 [2]
	P. menispermifolia	JQ588581 [2]
	P. murucuja	AY271612 [1]
	P. ornithoura	AY271613 [1]
	P. platyloba	JQ588582 [2]
		KJ751095 [10]
	P. quadrangularis	AB233808 [11]
		FM179937 [12]
		GQ248176 [2]
		KJ751079–KJ751081 [10]
		KJ751085 [10]
		KJ751087 [10]
		KJ751090 [10]
		KJ751092 [10]
		KJ751096–KJ751100 [10]
	P. sexflora	AY271614 [1]
	P. suberosa	DQ401363 [13]
		GU266608 [14]
	P. talamancensis	AY271615 [1]
	P. tetrandra	AB536650 [3]
	P. tulae	AY271616 [1]

Table S3. *Cont.*

Plastid Marker	Species	GenBank Access
rbcL	*P. actinia*	DQ123347 [15]
		HQ900845 [16]
	P. alata	DQ123348 [15]
		HQ900846 [16]
	P. ambigua	DQ123349 [15]
		JQ593081–JQ593084 [17]
	P. amoena	DQ123301 [15]
	P. antioquiensis	DQ123342 [15]
	P. arborea	DQ123300 [15]
	P. aurantioides	AB536553 [18]
	P. auriculata	DQ445921 [15]
		HQ900847 [16]
	P. bicornis	JQ593085–JQ593087 [17]
	P. biflora	EU017122 [19]
		GU135279 [20]
		JQ593088– JQ593092 [17]
	P. caerulea	DQ123350 [15]
		HM850239 [21]
		HQ900848 [16]
	P. campanulata	DQ123339 [15]
		HQ900849 [16]
	P. candida	DQ123302 [15]
	P. capparidifolia	HQ900850 [16]
	P. capsularis	DQ123312 [15]
		HQ900851 [16]
	P. cerasina	HQ900852 [16]
	P. ceratocarpa	DQ123303 [15]
	P. cerradensis	HQ900853 [16]
	P. ciliata	JX664062 [22]
	P. cincinnata	DQ123351 [15]
	P. cirrhiflora	DQ123377 [15]
	P. citrifolia	DQ123304 [15]
	P. clathrata	DQ123336 [15]
	P. coccinea	DQ123333 [15]
		HQ900854 [16]
	P. coriacea	DQ123313 [15]
	P. costaricensis	JQ593093–JQ593094 [17]
	P. cuprea	DQ123378 [15]
	P. deidamioides	DQ445925 [15]
		HQ900855 [16]
	P. edmundoi	DQ123352 [15]
		HQ900856 [16]

Table S3. *Cont.*

Plastid Marker	Species	GenBank Access
rbcL	*P. edulis*	DQ123353 [15]
		GQ436714 [23]
		HG765072 [24]
		HQ900857 [16]
	P. eichleriana	DQ123354 [15]
		HQ900858 [16]
	P. elegans	DQ123355 [15]
	P. exura	DQ123356 [15]
	P. foetida	DQ123337 [15]
		HQ900859 [16]
		KF425764 [25]
	P. gabrielliana	DQ123357 [15]
	P. galbana	DQ123358 [15]
		HQ900860 [16]
	P. garckei	DQ123359 [15]
	P. gardneri	HQ900861 [16]
	P. gilbertii	DQ445922 [15]
		HQ900862 [16]
	P. haematostigma	DQ123305 [15]
	P. hatschbachii	HQ900863 [16]
	P. helleri	DQ123314 [15]
	P. incarnata	DQ123360 [15]
		EF590556 [26]
		GQ248664 [27]
		HF565321 [28]
		HG765070–HG765071 [24]
		HQ900864 [16]
	P. iodocarpa	HQ900865 [16]
	P. ishnoclada	HQ900866 [16]
	P. jilekii	DQ123361 [15]
		HQ900867 [16]
	P. kawensis	DQ123306 [15]
	P. lancetillensis	DQ123331 [15]
	P. leptoclada	DQ445923 [15]
		HQ900869 [16]
	P. ligularis	HQ900870 [16]
	P. lindeniada	DQ123307 [15]
	P. lobbii subsp. ayaucuchoensis	DQ123315 [15]
	P. lobbii subsp. obtusiloba	DQ123316 [15]
	P. loefgrenii	HQ900871 [16]
	P. luetzelburgii	DQ123384 [15]
	P. lutea	DQ006111 [29]
	P. macrophylla	DQ123308 [15]
	P. maliformis	DQ123362 [15]
	P. manicata	DQ123344 [15]

Table S3. *Cont.*

Plastid Marker	Species	GenBank Access
rbcL	*P. mansoi*	DQ123309 [15]
	P. mathewsii	DQ123380 [15]
	P. mendoncaei	DQ123385 [15]
	P. menispermifolia	JQ593095–JQ593096 [17]
	P. micropetala	DQ445924 [15]
		HQ900872 [16]
	P. microstipula	DQ123332 [15]
	P. miersii	DQ123363 [15]
		HQ900873 [16]
	P. misera	DQ123317 [15]
		HQ900874 [16]
	P. mixta	DQ123381 [15]
	P. morifolia	DQ123318 [15]
		HQ900875 [16]
	P. mucronata	HQ900876 [16]
	P. multiflora	DQ123297 [15]
	P. murucuja	DQ123345 [15]
	P. nitida	DQ123364 [15]
		HQ900878 [16]
	P. odontophylla	DQ123365 [15]
	P. organensis	DQ123319 [15]
		HQ900877 [16]
	P. ornithoura	DQ123320 [15]
	P. ovalis	DQ123401 [15]
	P. palmeri	DQ123338 [15]
		HQ900879 [16]
	P. penduliflora	DQ123298 [15]
	P. picturata	HQ900880 [16]
	P. pilosicorona	HQ900881 [16]
	P. pittieri	DQ123310 [15]
	P. platyloba	HQ900882 [16]
		JQ593097–JQ593099 [17]
	P. pohlii	DQ123321 [15]
		HQ900883 [16]
	P. punctata	DQ123322 [15]
	P. quadrangularis	AB233912 [30]
		DQ123366 [15]
		EF590557 [26]
		GQ248665 [27]
		L01940 [31]
	P. racemosa	DQ123311 [15]
		HQ900884 [16]
	P. recurva	DQ123367 [15]
	P. reflexiflora	DQ123386 [15]
	P. rhamnifolia	DQ123299 [15]

Table S3. *Cont.*

Plastid Marker	Species	GenBank Access
rbcL	*P. riparia*	DQ123368 [15]
	P. rufa	DQ123323 [15]
	P. serratifolia	DQ123369 [15]
	P. serratodigitata	DQ123370 [15]
		HQ900885 [16]
	P. setacea	DQ123371 [15]
	P. setulosa	DQ123340 [15]
	P. sexflora	DQ123324 [15]
	P. sidiifolia	DQ123372 [15]
		HQ900886–HQ900887 [16]
	P. speciosa	DQ123334 [15]
	P. sprucei	DQ123373 [15]
	P. suberosa	DQ123325 [15]
		HQ900888 [16]
	P. subrotunda	HQ900889 [16]
	P. tacsonioides	DQ123379 [15]
	P. talamancensis	DQ123326 [15]
	P. tenuifila	DQ123374 [15]
	P. tetrandra	AB536572 [18]
	P. tricuspis	DQ123327 [15]
		HQ900890 [16]
	P. trifasciata	DQ123328 [15]
	P. trifoliata	DQ123383 [15]
	P. trintae	DQ123375 [15]
	P. tripartita	DQ123382 [15]
	P. trisecta	DQ123343 [15]
	P. truncata	HQ900891 [16]
	P. tryphostemmatoides	DQ123388 [15]
	P. tulae	DQ123346 [15]
		HQ900892 [16]
	P. umbilicata	DQ123387 [15]
	P. urubiciensis	HQ900893 [16]
	P. vespertilio	DQ123329 [15]
		HQ900894 [16]
	P. villosa	DQ123341 [15]
	P. vitifolia	DQ123335 [15]
		HQ900895 [16]
		JQ593100–JQ593102 [17]
	P. watsoniana	DQ123376 [15]
		HQ900896 [16]
	P. xiikzodz	DQ123330 [15]

Table S3. *Cont.*

Plastid Marker	Species	GenBank Access
trnH-psbA	*P. actinia*	AY032807 [32]
		AY219288–AY219299 [33]
	P. alata	AY032808 [32]
	P. biflora	GU135451 [34]
	P. caerulea	AY032816 [32]
		AY220135 [35]
	P. campanulata	AY032812 [32]
	P. capsularis	AY032822 [32]
	P. cincinnata	DQ238756 [36]
	P. coriacea	DQ238763 [36]
	P. edulis	AY032811 [32]
	P. elegans	AY032806 [32]
		AY219300–AY219310 [33]
	P. foetida	AY032814 [32]
		DQ238759 [36]
		AY220136 [35]
	P. galbana	AY032817 [32]
		AY220137 [35]
	P. haematostigma	AY032819 [32]
	P. incarnata	EF590722 [37]
		GQ248361 [38]
		DQ238757 [36]
		AY032810 [32]
	P. jilekii	AY220138 [35]
	P. kermesina	AY032815 [32]
	P. lutea	DQ006208 [39]
	P. misera	AY032804 [32]
	P. morifolia	AY032805 [32]
	P. organensis	AY032803 [32]
	P. palmeri	DQ249919 [36]
	P. pohlii	AY032802 [32]
	P. quadrangularis	EF590723 [37]
		AY032809 [32]
		GQ248362 [38]
	P. rubra	AY032821 [32]
	P. setulosa	AY032818 [32]
	P. sidiifolia	AY220139 [35]
	P. sprucei	DQ249920 [36]
	P. suberosa	AY032820 [32]
	P. tenuifila	AY032813 [32]
		AY220140 [35]
	P. xiikzodz	DQ238762 [36]

Table S3. *Cont.*

Plastid Marker	Species	GenBank Access
trnL (UAA) intron	*P. actinia*	HQ900949 [40]
		DQ123065 [41]
	P. acuminata	DQ123066 [41]
	P. adenopoda	AY632727 [42]
	P. alata	AF454778 [43]
		HQ900950 [40]
		DQ123067 [41]
	P. alnifolia	JX470862 [44]
	P. ambigua	AF454779 [43]
		DQ123068 [41]
	P. amethystina	DQ123069 [41]
	P. amoena	DQ123017 [41]
	P. ampullacea	AY632745 [42]
	P. anadenia	JX470863 [44]
	P. antioquiensis	DQ123060 [41]
	P. apoda	JX470864 [44]
	P. arbelaezii	AY632728 [42]
	P. arborea	JX470865 [44]
		DQ123018 [41]
	P. aurantia	AY632729 [42]
	P. auriculata	AF454780 [43]
		HQ900951 [40]
		DQ284534 [45]
	P. bicornis	JX470866 [44]
	P. biflora	AF454781 [43]
		JX470867 [44]
		AY632730 [42]
	P. boenderi	JX470868 [44]
	P. bryonioides	JX470869 [44]
	P. caerulea	AF454784 [43]
		HQ900952 [40]
		DQ123070 [41]
	P. campanulata	HQ900953 [40]
		DQ123057 [41]
	P. candida	DQ123019 [41]
	P. capparidifolia	HQ900954 [40]
	P. capsularis	HQ900955 [40]
		DQ123029 [41]
	P. cerasina	HQ900956 [40]
	P. ceratocarpa	DQ123020 [41]
	P. cerradensis	HQ900957 [40]
	P. cf. *viridescens*	JX470914 [44]
	P. chelidonea	JX470870–JX470871 [44]
	P. chrysosepala	JX470872 [44]
	P. cincinnata	DQ123071 [41]

Table S3. *Cont.*

Plastid Marker	Species	GenBank Access
trnL (UAA) intron	*P. cinnabarina*	AY632731 [42]
	P. cirrhiflora	DQ123101 [41]
	P. citrifolia	AY632732 [42]
		DQ123021 [41]
	P. clathrata	DQ123054 [41]
	P. cobanensis	JX470873 [44]
	P. coccinea	HQ900958 [40]
	P. contracta	KF196437–KF196509 [46]
	P. coriacea	AF454782 [43]
		DQ123030 [41]
	P. cubensis	JX470875 [44]
	P. cupiformis	AY632733 [42]
	P. cupraea	JX470876 [44]
		DQ123102 [41]
	P. deidamioides	HQ900959 [40]
	P. eberhartii	JX470877 [44]
	P. edmundoi	HQ900960 [40]
		DQ123072 [41]
	P. edulis	AF454783 [43]
		HQ900961 [40]
		JX470878 [44]
		DQ123073 [41]
	P. eichleriana	HQ900962 [40]
		DQ123074 [41]
	P. elegans	DQ123075 [41]
	P. escobariana	JX470879 [44]
	P. exsudans	JX470880 [44]
	P. exura	DQ123076 [41]
	P. filipes	AY632734 [42]
	P. foetida	HQ900963 [40]
		JQ723387 [47]
		DQ123055 [41]
	P. gabrielliana	DQ123077 [41]
	P. galbana	HQ900964 [40]
		DQ123078 [41]
	P. garckei	DQ123079 [41]
	P. gardineri	HQ900965 [40]
	P. gilbertii	HQ900966 [40]
	P. gilbertiana	JX470881 [44]
	P. gracillima	JX470882 [44]
		DQ458091 [48]
	P. guatemalensis	JX470883 [44]
	P. haematostigma	DQ123022 [41]
	P. hatschbachii	HQ900967 [40]
	P. helleri	DQ123031 [41]

Table S3. *Cont.*

Plastid Marker	Species	GenBank Access
trnL (UAA) intron	*P. henryi*	AY632735 [42]
	P. herbertiana	AY632736 [42]
	P. hirtiflora	JX470885 [44]
	P. ichthyura	JX470886 [44]
	P. incarnata	AY756890 [49]
		HQ900968 [40]
		DQ123080 [41]
	P. indecora	JX470938 [44]
	P. iodocarpa	HQ900969 [40]
	P. ischnoclada	HQ900970 [40]
		DQ123081 [41]
	P. jilekii	HQ900971 [40]
		DQ123082 [41]
	P. jugorum	AY632737 [42]
	P. jussieu	JX470943 [44]
	P. karwinskii	JX470887 [44]
	P. kawensis	DQ123023 [41]
	P. kermesina	HQ900972 [40]
		DQ123083 [41]
	P. lancearia	JX470888 [44]
	P. lancetillensis	DQ123050 [41]
	P. leptoclada	HQ900973 [40]
		JX470889 [44]
	P. ligularis	HQ900974 [40]
	P. lindeniana	DQ123024 [41]
	P. lobata	AF454787 [43]
	P. lobbii ayaucuchoensis	DQ123032 [41]
	P. lobbii obtusiloba	DQ123033 [41]
	P. loefgrenii	HQ900975 [40]
	P. luetzerburgii	DQ123109 [41]
	P. lutea	JX470890 [44]
	P. macrophylla	DQ123025 [41]
	P. maestrensis	JX470891 [44]
	P. maliformis	DQ123025 [41]
	P. manicata	DQ123062 [41]
	P. mansoi	DQ123026 [41]
	P. mathewsii	DQ123105 [41]
	P. membranacea	AY632726 [42]
	P. mendoncaei	DQ123110 [41]
	P. menispermifolia	AF454785 [43]
	P. mexicana	AY632738 [42]
	P. micropetala	HQ900976 [40]
	P. microstipula	DQ123051 [41]
	P. miersii	HQ900977 [40]
		DQ123085 [41]

Table S3. *Cont.*

Plastid Marker	Species	GenBank Access
trnL (UAA) intron	*P. misera*	HQ900978 [40]
		JX470892 [44]
		DQ123034 [41]
	P. mixta	DQ123106 [41]
	P. molissima	AF454788 [43]
	P. moluccana	AY632739 [42]
	P. morifolia	HQ900979 [40]
		DQ123035 [41]
	P. mucronata	HQ900980 [40]
		DQ123086 [41]
	P. multiflora	AY632740 [42]
		DQ123014 [41]
	P. murucuja	JX470894 [44]
		AY632747 [42]
		DQ123063 [41]
	P. nitida	HQ900982 [40]
		DQ123087 [41]
	P. oblongata	JX470895 [44]
	P. obtusifolia	JX470896 [44]
	P. odontophylla	DQ123088 [41]
	P. oerstedii	AF454786 [43]
	P. organensis	HQ900981 [40]
		DQ123036 [41]
	P. ornithoura	DQ123037 [41]
	P. ovalis	DQ123122 [41]
		KF196419–KF196436 [46]
	P. palmeri	HQ900983 [40]
		DQ123056 [41]
	P. penduliflora	JX470898 [44]
		DQ123015 [41]
	P. perfoliata	JX470899 [44]
	P. picturata	HQ900984 [40]
	P. pilosicorona	HQ900985 [40]
	P. pittieri	AF454789 [43]
		DQ123027 [41]
	P. platyloba	AF454790 [43]
		HQ900986 [40]
	P. podlechii	DQ123013 [41]
	P. pohlii	HQ900987 [40]
		DQ123038 [41]
	P. porphyretica	JX470939 [44]
	P. punctata	DQ123039 [41]
	P. pusilla	JX470900 [44]
	P. pyrrhantha	JX470901 [44]

Table S3. *Cont.*

Plastid Marker	Species	GenBank Access
trnL (UAA) intron	*P. quadrangularis*	AF454791 [43]
		DQ123089 [41]
	P. racemosa	HQ900988 [40]
		DQ123028 [41]
	P. recurva	DQ123090 [41]
	P. reflexiflora	DQ123111 [41]
	P. rhamnifolia	DQ123016 [41]
	P. riparia	DQ123091 [41]
	P. rovirosae	DQ123040 [41]
	P. rubra	AY632741 [42]
	P. rufa	JX470902 [44]
		DQ123041 [41]
	P. rugosissima	JX470903 [44]
	P. sagasteguii	JX470904 [44]
	P. sandrae	JX470940 [44]
	P. sanguinolenta	JX470905 [44]
		DQ123104 [41]
	P. serratifolia	DQ123092 [41]
	P. serratodigitata	HQ900989 [40]
		DQ123093 [41]
	P. setacea	DQ123094 [41]
	P. setulosa	DQ123058 [41]
	P. sexflora	JX470906 [44]
		DQ123042 [41]
	P. siamica	AY632742 [42]
	P. sidiifolia	HQ900990–HQ900991 [40]
		DQ123095 [41]
	P. sodiroi	JX470907 [44]
	P. solomonii	JX470908 [44]
	P. speciosa	DQ123052 [41]
	P. sphaerocarpa	JX470909 [44]
	P. sprucei	DQ123096 [41]
	P. suberosa	AF454792 [43]
		HQ900992 [40]
		AY632743 [42]
		DQ123043 [41]
	P. subrotunda	HQ900993 [40]
	P. tacanensis	JX470910 [44]
	P. tacsonioides	DQ123103 [41]
	P. talamancensis	AF454793 [43]
		DQ123044 [41]
	P. tatei	JX470941 [44]
	P. tenuifila	DQ123097 [41]
	P. tenuiloba	AY632744 [42]
	P. tetrandra	AY632746 [42]

Table S3. *Cont.*

Plastid Marker	Species	GenBank Access
trnL (UAA) intron	*P. tica*	AF461415 [43]
	P. tina	JX470911 [44]
	P. tricuspis	HQ900994 [40]
		DQ123045 [41]
	P. trifasciata	DQ123046 [41]
	P. trifoliata	DQ123108 [41]
	P. trintae	DQ123098 [41]
	P. tripartita	DQ123107 [41]
	P. trisecta	DQ123061 [41]
	P. truncata	HQ900995 [40]
		DQ123047 [41]
	P. tryphostemmatoides	DQ123113 [41]
	P. tulae	HQ900996 [40]
		JX470912 [44]
		DQ123064 [41]
	P. umbilicata	DQ123112 [41]
	P. urnifolia	JX470942 [44]
	P. urubiciensis	HQ900997 [40]
		DQ123099 [41]
	P. vespertilio	HQ900998 [40]
		JX470913 [44]
		DQ123048 [41]
	P. villosa	DQ123059 [41]
	P. vitifolia	AF454794 [43]
		HQ900999 [40]
		JX470915 [44]
		DQ123053 [41]
	P. watsoniana	HQ901000 [40]
		DQ123100 [41]
	P. xiikzodz	JX470916 [44]
		DQ123049 [41]

1: Yockteng R, Nadot S (2004) Infrageneric phylogenies: a comparison of chloroplast-expressed glutamine synthetase, cytosol-expressed glutamine synthetase and cpDNA maturase K in *Passiflora*. Molecular Phylogenetics and Evolution, 31, 397-402; 2: International Barcode of Life (iBOL). Unpublished; 3: Tokuoka T. Molecular phylogenetic analysis of Passifloraceae *sensu lato* (Malpighiales) based on plastid and nuclear DNA sequences. Unpublished; 4: Jansen RK, Cai Z, Raubeson LA, *et al.* (2007) Analysis of 81 genes from 64 plastid genomes resolves relationships in angiosperms and identifies genome-scale evolutionary patterns. Proceedings of the National Academy of Sciences of United States of America. 104, 19369-19374; 5: Abbott JR, Neubig KM, Whitten WM, Williams NH. DNA barcoding the flora of Florida: Invasive species. Unpublished; 6: Schaefer H, Hardy OJ, Silva L, Barraclough TG, Savolainen V (2011). Testing Darwin's naturalization hypothesis in the Azores. Ecol. Lett. 14, 389-396; 7: Roeder M, Slik JWF. Phylogenetic structure of liana communities along a disturbance gradient in South West China. Unpublished; 8: Xi Z, Ruhfel BR, Schaefer H, *et al.* (2012) Phylogenomics and a posteriori data partitioning resolve the Cretaceous angiosperm radiation Malpighiales. Proc. Natl. Acad. Sci. U.S.A. 109, 17519-17524; 9: Davis CC, LatvisM, Nickrent DL, Wurdack KJ, Baum DA (2007) Floral gigantism in Rafflesiaceae.

Science 315 (5820), 1812; 10: Palhares RM. Barcode identification of medicinal plants and comparison with chemical identification. Unpublished; 11: Tokuoa T, Tobe H (2006) Phylogenetic analyses of Malpighiales using plastid and nuclear DNA sequences, with particular reference to the embryology of Euphorbiaceae sens. Str. J. Plant Res. 119, 599-616; 12: Worberg A, Alford MH, Quandt D, Borsch T (2009) Huerteales sister to Brassicales plus Malvales, and newly circumscribed to include Dipentodon, Gerrardina, Huertea, Perrottetia, and Tapiscia. Taxon 58, 468-478; 13: Qiu YL, Li L, Hendry TA *et al*. Reconstructing the Basal Angiosperm Phylogeny: Evaluating Information Content of the Mitochondrial Genes. Unpublished; 14: Hilu KW, Black C, Diouf D, Burleigh JG (2008) Phylogenetic signal in matK vs. trnK: a case study in early diverging eudicots (angiosperms). Mol. Phylogenet. Evol. 48, 1120-1130; 15: Muschner VC, Lorenz-Lemke AP, Cervi AC, Bonatto S, Freitas LB. Phylogenetic relationships among *Passiflora* (Passifloraceae) species: a new taxonomic proposal. Unpublished; 16: Yotoko KS, Dornelas MC, Togni PD, *et al*. (2011) Does variation in genome sizes reflect adaptive or neutral processes? New clues from *Passiflora*. PLoS ONE 6: E18212; 17: International Barcode of Life (iBOL). Unpublished; 18: Tokuoka T. Molecular phylogenetic analysis of Passifloraceae *sensu lato* (Malpighiales) based on plastid and nuclear DNA sequences. Unpublished; 19: Jansen RK, Cai Z, Raubeson LA, *et al*. (2007) Analysis of 81 genes from 64 plastid genomes resolves relationships in angiosperms and identifies genome-scale evolutionary patterns. Proceedings of the National Academy of Sciences of United States of America. 104, 19369-19374; 20: Abbott JR, Neubig KM, Whitten WM, Williams NH. DNA barcoding the flora of Florida: Invasive species. Unpublished; 21: Schaefer H, Hardy OJ, Silva L, Barraclough TG, Savolainen V (2011). Testing Darwin's naturalization hypothesis in the Azores. Ecol. Lett. 14, 389-396; 22: Xi Z, Ruhfel BR, Schaefer H, *et al*. (2012) Phylogenomics and a posteriori data partitioning resolve the Cretaceous angiosperm radiation Malpighiales. Proc. Natl. Acad. Sci. U.S.A. 109, 17519-17524; 23: Chen S, Yao H, Han J, *et al*. (2010) Validation of the ITS2 region as a novel DNA barcode for identifying medicinal plant species. PLoS ONE 5, E8613; 24: Laiou A, Mandolini LA, Piredda R, Bellarosa R, Simeone MC (2013) DNA barcoding as a complementary tool for conservation and valorisation of forest resources. Zookeys 365, 197-213; 25: Saritha KV, Khedar GD, Hanumanth Kumar G, Tiknaik AD, Ughade BR. Direct submission; 26: Kress J, Erickson DL (2007) A two-locus global DNA barcode for land plants: the coding rbcL gene complements the non-coding trnH-psbA spacer region. PLoS ONE 2, E508; 27: CBOL Plant Working Group. A DNA Barcode for Land Plants. Unpublished; 28: Cornara L, Borghesi B, Canali C, *et al*. (2013) Smart drugs: green shuttle or real drug? Int. J. Legal Med. 127, 1109-1123; 29: Kress WJ, Wurdack KJ, Zimmer EA, Weigt LA, Janzen DH (2005). Use of DNA barcodes to identify flowering plants. Proc. Natl. Acad. Sci. U.S.A. 102, 8369-8374; 30: Tokuoa T, Tobe H (2006) Phylogenetic analyses of Malpighiales using plastid and nuclear DNA sequences, with particular reference to the embryology of Euphorbiaceae sens. Str. J. Plant Res. 119, 599-616; 31: Albert VA, Williams SE, Chase MW (1992) Carnivorous plants: phylogeny and structural evolution. Science 257 (5076), 1491-1495 (1992); 32: Muschner VC, Lorenz AP, Scherer NM, *et al*. Comparative Phylogenetic Analysis of Nuclear and Plastid Sequences in *Passiflora* (Passifloraceae). Unpublished; 33: Lorenz-Lemke AP, Muschner VC, Bonatto SL, Cervi AC, Salzano FM, Freitas LB (2006) Phylogeographic inferences concerning evolution of Brazilian *Passiflora actinia* and *P. elegans* (Passifloraceae) based on ITS (nrDNA) variation. Ann. Bot. 95: 799-806; 34: Abbott JR, Neubig KM, Whitten WM, Williams NH. DNA barcoding the flora of Florida: Invasive species. Unpublished; 35: Lorenz AP, Muschner VC, Bonatto SL, Salzano FM, Freitas LB. Molecular evidence for the origin of *Passiflora elegans* (Passifloraceae) from southern Brazil Unpublished; 36: Muschner VC, Lorenz-Lemke AP, Vecchia M, Bonatto SL, Salzano FM, Freitas LB (2006) Differential organellar inheritance in *Passiflora* (Passifloraceae) subgenera. Genetica 128, 449-453; 37: Kress J, Erickson DL (2007) A two-locus global DNA barcode for land plants: the coding rbcL gene complements the non-coding trnH-psbA spacer region. PLoS ONE 2, E508; 38: CBOL Plant Working Group. A DNA Barcode for Land Plants. Unpublished; 39: Kress WJ, Wurdack KJ, Zimmer EA, Weigt LA, Janzen DH (2005). Use of DNA barcodes to identify

flowering plants. Proc. Natl. Acad. Sci. U.S.A. 102, 8369-8374; 40: Yotoko KS, Dornelas MC, Togni PD, *et al*. (2011) Does variation in genome sizes reflect adaptive or neutral processes? New clues from *Passiflora*. PLoS ONE 6: E18212; 41: Muschner VC, Lorenz-Lemke AP, Cervi AC, Bonatto S, Freitas LB. Phylogenetic relationships among *Passiflora* (Passifloraceae) species: a new taxonomic proposal. Unpublished; 42: Krosnick SE, Freudenstein JV (2005) Monophyly and floral character homology of old world *Passiflora* (Subgenus *Decaloba*: Supersection *Disemma*). Systematic Botany, 30, 139-152; 43: Ossowski AM, Hunter FF. Coevolution of *Heliconius* spp. and *Passiflora* spp.: A phylogenetic comparison. Unpublished; 44: Krosnick SE, Porter-Utley KE, MacDougal JM, Jørgensen PM, McDade LA (2013) New insights into the evolution of *Passiflora* subgenus *Decaloba* (Passifloraceae): phylogenetic relationships and morphological synapomorphies. Systematic Botany, 38, 692-713; 45: Krosnick SE, Freudenstein JV (2006) Patterns of anomalous floral development in the Asian *Passiflora* (subgenus *Decaloba*: supersection *Disemma*). Am. J. Bot., 93, 620-636; 46: Cazé ALR, Mäder G, Bonatto SL, Freitas LB (2013) A molecular systematic analysis of *Passiflora ovalis* and *Passiflora contracta* (Passifloraceae). Phytotaxa, 132, 39-46; 47: Thulin M, Razafimandimbison SG, Chafe P, Heidari N, Kool A, Shore JS (2012) Phyloheny of the Turneracea clade (Passifloraceae): Trans-Atlantic disjunctions and two new genera in Africa. Taxon, 61, 308-323; 48: Krosnick SE, Ford A, Freudenstein JV. Resolving the phylogenetic position of *Hollrungia* and *Tetrapathaea*: The end of two monotypic genera in Passifloraceae. Unpublished; 49: Alford, MH. Phylogeny, character evolution, and classification of the Flacourtiaceae/Salicaceae complex. Unpublished.

Table S4. Primer sequences and references for studied ITS sequences.

Article Reference	Primers Used for ITS Sequences	Primer Reference
Cazé, A.L.R.; Mäder, G.; Bonatto, S.L.; Freitas, L.B. A molecular systematic analysis of *Passiflora ovalis* and *Passiflora contracta* (Passifloraceae). *Phytotaxa* **2013**, *132*, 39–46.	5' AAGGTTTCCGTAGGTGAAC 3' and 5' TATGCTTAAACTCAGCGGG 3'	Desfeux & Lejeune (1996)
Giudicelli *et al*. (in prep)	5' AAGGTTTCCGTAGGTGAAC 3' and 5' TATGCTTAAACTCAGCGGG 3'	Desfeux & Lejeune (1996)
Hearn, D.J. Adenia (Passifloraceae) and its adaptative radiation: Phylogeny and growth form diversification. *Syst. Bot.* **2006**, *31*, 805–821.	N18S (5' AGGAGAAGTCGTAACAGG 3') and C26A (5' GTTTCTTTTCCTCCGCT 3')	Modified from Wen and Zimmer (1996)
Kay, E.E. Floral Evolutionary Ecology of Passiflora: subgenera Murucuia, Pseudomurucuja and Astephia. Unpublished.	GenBank information: unpublished	
Koehler-Santos, P.; Lorenz-Lemke, A.P.; Muschner, V.C.; Bonatto, S.L.; Salzano, F.M.; Freitas, L.B. Molecular genetic variation in *Passiflora alata* (Passifloraceae), na invasive species in southern Brazil. Biological Journal of the Linnean *Society* **2006**, *88*, 611–630.	5' AAGGTTTCCGTAGGTGAAC 3' and 5' TATGCTTAAACTCAGCGGG 3'	Desfeux & Lejeune (1996)
Kress, W.J.; Wurdack, K.J.; Zimmer, E.A.; Weigt, L.A; Janzen, D.H. Use of DNA barcodes to identify flowering plants. *Proc. Natl. Acad. Sci. USA* **2005**, *102*, 8369–8374.	Primers 5 (5' GGAAGTAAAAGTCGTAACAAGG 3') and 4 (5' TCCTCCGCTTATTGATATGC 3')	White *et al*. (1990)
Krosnick SE, Ford A, Freudenstein JV. Resolving the phylogenetic position of *Hollrungia* and *Tetrapathaea*: The end of two monotypic genera in Passifloraceae. Unpublished.	GenBank information: unpublished	
Krosnick, S.E.; Freudenstein, J.V. Monophyly and floral character homology of old world *Passiflora* (Subgenus *Decaloba*: Supersection *Disemma*). *Syst. Bot.* **2005**, *30*, 139–152.	Primers 5 (5' GGAAGTAAAAGTCGTAACAAGG 3') and 4 (5' TCCTCCGCTTATTGATATGC 3')	White *et al*. (1990)

Table S4. *Cont.*

Article Reference	Primers Used for ITS Sequences	Primer Reference
Krosnick, S.E.; Freudenstein, J.V. Patterns of anomalous floral development in the Asian *Passiflora* (Subgenus *Decaloba*: Supersection *Disemma*). *Am. J. Bot.* **2006**, *93*, 620–636.	Primers 5 (5' GGAAGTAAAAGTCGTAACAAGG 3') and 4 (5' TCCTCCGCTTATTGATATGC 3')	White *et al*. (1990)
Krosnick, S.E.; Freudenstein, J.V. Phylogenetic relationships among the Old World species of *Passiflora* L. (Subgenus *Decaloba*: Supersection *Disemma*). Unpublished.	GenBank information: unpublished	
Krosnick, S.E.; Porter-Utley, K.E.; MacDougal, J.M.; Jørgensen, P.M.; McDade, L.A. New insights into the evolution of *Passiflora* subgenus *Decaloba* (Passifloraceae): Phylogenetic relationships and morphological synapomorphies. *Syst. Bot.* **2013**, *38*, 692–713.	Primers 5 (5' GGAAGTAAAAGTCGTAACAAGG 3') and 4 (5' TCCTCCGCTTATTGATATGC 3')	White *et al*. (1990)
Krosnick, S.E.; Xun-Lin, Y.; Deng, Y. The rediscovery of *Passiflora kwangtungensis* Merr. (subgenus *Decaloba* supersection *Disemma*): A critically endangered Chinese endemic. *PhytoKeys* **2013**, *23*, 55–74.	Primers 5 (5' GGAAGTAAAAGTCGTAACAAGG 3') and 4 (5' TCCTCCGCTTATTGATATGC 3')	White *et al*. (1990)
Lorenz-Lemke, A.P.; Muschner, V.C.; Bonatto, S.L.; Cervi, A.C.; Salzano, F.M.; Freitas, L.B. Phylogeographic inferences concerning evolution of Brazilian *Passiflora actinia* and *P. elegans* (Passifloraceae) based on ITS (nrDNA) variation. *Ann. Bot.* **2005**, *95*, 799–806.	5' AAGGTTTCCGTAGGTGAAC 3' and 5' TATGCTTAAACTCAGCGGG 3'	Desfeux & Lejeune (1996)
Mäder, G.; Zamberlan, P.M.; Fagundes, N.J.R.; Magnus, T.; Salzano, F.M.; Bonatto, S.L.; Freitas, L.B. The use and limits of ITS data in the analysis of intraspecific variation in *Passiflora* L. (Passifloraceae). *Genet. Mol. Biol.* **2010**, *33*, 99–108.	5' AAGGTTTCCGTAGGTGAAC 3' and 5' TATGCTTAAACTCAGCGGG 3'	Desfeux & Lejeune (1996)
Muschner, V.C.; Lorenz, A.P.; Cervi, A.C.; Bonatto, S.L.; Souza-Chies, T.T.; Salzano, F.M.; Freitas, L.B. A first molecular phylogenetic analysis of *Passiflora* (Passifloraceae). *Am. J. Bot.* **2003**, *90*, 1229–1238.	5' AAGGTTTCCGTAGGTGAAC 3' and 5' TATGCTTAAACTCAGCGGG 3'	Desfeux & Lejeune (1996)

Table S4. *Cont.*

**Article Reference**	**Primers Used for ITS Sequences**	**Primer Reference**
Muschner, V.C.; Lorenz-Lemke, A.P.; Vecchia, M.; Bonatto, S.L.; Salzano, F.M.; Freitas, L.B. Differential organellar inheritance in *Passiflora* (Passifloraceae) subgenera. *Genetica* **2006**, *128*, 449–453.	5' AAGGTTTCCGTAGGTGAAC 3' and 5' TATGCTTAAACTCAGCGGG 3'	Desfeux & Lejeune (1996)
Ossowski, A.M.; Hunter, F.F. Coevolution of *Heliconius* spp. and *Passiflora* spp.: A phylogenetic comparison. Unpublished.	GenBank information: unpublished	
Thulin M, Razafimandimbison, S.G.; Chafe, P.; Heidari, N.; Kool, A.; Shore, J.S. Phyloheny of the Turneracea clade (Passifloraceae): Trans-Atlantic disjunctions and two new genera in Africa. *Taxon* **2012**, *61*, 308–323.	P17F (5' CTACCGATTGAATGGTCCGGTGAA 3') and 26S–82R (5' TCCCGGTTCGCTCGCCGTTACTA 3')	Alejandro *et al*. (2005)
Wright, S.; Keeling, J.; Gillman, L. The road from santa Rosalia: A faster tempo of evolution on tropical climes. *Proc. Natl. Acad. Sci. USA* **2006**, *103*, 7718–7722.	CY1 (5' TACCGATTGAATGATCCGGTGAAG 3') and CY3 (5' CGCCGTTACTAGGGGAATCCTTGT 3')	C. G. Yong, personal communication
